# Role of PKR and Type I IFNs in Viral Control during Primary and Secondary Infection

**DOI:** 10.1371/journal.ppat.1000966

**Published:** 2010-06-24

**Authors:** Yumi Nakayama, Erin H. Plisch, Jeremy Sullivan, Chester Thomas, Charles J. Czuprynski, Bryan R. G. Williams, M. Suresh

**Affiliations:** 1 Department of Pathobiological Sciences, University of Wisconsin-Madison, Madison, Wisconsin, United States of America; 2 Monash Institute of Medical Research, Monash University, Clayton, Victoria, Australia; University of North Carolina, United States of America

## Abstract

Type I interferons (IFNs) are known to mediate viral control, and also promote survival and expansion of virus-specific CD8^+^ T cells. However, it is unclear whether signaling cascades involved in eliciting these diverse cellular effects are also distinct. One of the best-characterized anti-viral signaling mechanisms of Type I IFNs is mediated by the IFN-inducible dsRNA activated protein kinase, PKR. Here, we have investigated the role of PKR and Type I IFNs in regulating viral clearance and CD8^+^ T cell response during primary and secondary viral infections. Our studies demonstrate differential requirement for PKR, in viral control versus elicitation of CD8^+^ T cell responses during primary infection of mice with lymphocytic choriomeningitis virus (LCMV). PKR-deficient mice mounted potent CD8^+^ T cell responses, but failed to effectively control LCMV. The compromised LCMV control in the absence of PKR was multifactorial, and linked to less effective CD8^+^ T cell-mediated viral suppression, enhanced viral replication in cells, and lower steady state expression levels of IFN-responsive genes. Moreover, we show that despite normal expansion of memory CD8^+^ T cells and differentiation into effectors during a secondary response, effective clearance of LCMV but not vaccinia virus required PKR activity in infected cells. In the absence of Type I IFN signaling, secondary effector CD8^+^ T cells were ineffective in controlling both LCMV and vaccinia virus replication in vivo. These findings provide insight into cellular pathways of Type I IFN actions, and highlight the under-appreciated importance of innate immune mechanisms of viral control during secondary infections, despite the accelerated responses of memory CD8^+^ T cells. Additionally, the results presented here have furthered our understanding of the immune correlates of anti-viral protective immunity, which have implications in the rational design of vaccines.

## Author Summary

Type I interferons (IFNs) constitute the first line of defense against viral infections, promote antigen presentation by dendritic cells, and play a crucial role in directly stimulating anti-viral T cell responses. However, the mechanisms underlying the diverse cellular effects of Type I IFNs are not well defined. One of the best-characterized anti-viral signaling mechanisms induced by Type I IFNs is mediated by the IFN-inducible dsRNA activated protein kinase, PKR. We show that requirement for cellular PKR activity could be a distinguishing feature between Type I IFN actions that mediate viral control or stimulate CD8^+^ T cell expansion during an acute infection with lymphocytic choriomeningitis virus (LCMV). Typically, innate immune mechanisms including Type I IFNs are considered important for viral control during a primary infection. However, we find that presence of vaccine-induced CD8^+^ T cell memory and accelerated generation of secondary effectors are necessary but not sufficient to provide effective protective immunity to re-infection, without the aid of innate effectors PKR and Type I IFNs. These findings have improved our understanding of virus-immune system interactions and immune correlates of anti-viral protective immunity, which might have implications in the development of effective anti-viral vaccines and immunotherapies.

## Introduction

Innate immunity constitutes the first line of anti-microbial host defense and plays an important role in controlling the spread of pathogens, before the onset of adaptive immunity [1]. Innate immunity depends on immune cells such as macrophages, natural killer (NK) cells, dendritic cells (DCs) and the cytokines produced by them. Type I interferons (IFNs) are primary cytokines produced after viral infections, which induce an antiviral state in neighboring cells by upregulating transcription of IFN-stimulated genes (ISGs) [2], [3]. Moreover, Type I IFNs are known to promote CD8^+^ and CD4^+^ T cell responses by both direct and indirect effects [4], [5], [6], [7], [8]. However, the signaling mechanisms underlying the diverse cellular effects of Type I IFNs are not well understood.

One of the best-characterized anti-viral signaling mechanisms of Type I IFNs is mediated by the IFN-inducible dsRNA activated protein kinase, PKR [3], [9], [10]. PKR, an intracellular receptor for dsRNA, is expressed ubiquitously at low levels as an inactive kinase, and its transcription is upregulated by Type I IFN-signaling. Binding of dsRNA produced during viral replication alters the conformation of PKR, which leads to dimerization and activation by autophosphorylation. Once activated, PKR phosphorylates the α-subunit of eukaryotic initiation factor 2 (eIF-2α) to inhibit protein translation and suppress viral replication [11]. Not surprisingly, many viruses have evolved mechanisms to evade the anti-viral effects of PKR [12], [13], [14], [15]. In addition to the well-known anti-viral actions, by way of its effects on translation and transcription, PKR also regulates diverse processes including cellular differentiation, proliferation, and apoptosis [11], [16], [17]. In the immune system, PKR has been reported to mediate apoptosis of macrophages, suppress translational response in T cells, and downregulate T cell proliferation [18], [19], [20]. However, the role of PKR in regulating T cell activation, effector differentiation or function during an acute viral infection has not been investigated.

In addition to dsRNA and Type I IFNs, cellular PKR activation can be triggered by exposure to immune regulatory cytokines like IFN-γ and TNF-α [21], and full activation of cells in response to these cytokines is known to require PKR [22]. IFN-γ and TNF-α play non-redundant roles in governing the primary T cell responses to lymphocytic choriomeningitis virus (LCMV) [23], [24] but a role for PKR in regulation of anti-viral T cell responses has not been investigated.

The role of Type I IFNs in regulating anti-viral innate and adaptive immune responses has been extensively studied using the mouse model of LCMV. During a primary LCMV infection, Type I IFNs play a non-redundant role in effecting early viral control, and deficiency of Type I IFN receptor signaling leads to viral persistence [6], [25], [26]. Apart from their importance in innate immunity to LCMV, direct effects of Type I IFNs are required for activation and expansion of virus-specific CD8^+^ T cells during a primary response [5], [6]. Moreover, diverse effects of Type I IFNs on the activation and differentiation of dendritic cells during viral infection can indirectly regulate anti-viral T cell responses [27]. However, it is yet to be determined whether cellular effects of Type I IFNs in mediating LCMV control and/or CD8^+^ T cell activation during a primary infection include activation of PKR. Additionally, the requirement for Type I IFNs in controlling viral replication during secondary responses is unclear.

In this manuscript, we have determined: 1) the role of PKR in viral control versus regulation of virus-specific CD8^+^ T cell responses during primary and secondary LCMV infection; 2) the requirement for PKR in immune control of vaccinia virus in vaccinated mice; 3) whether Type I IFNs contribute to LCMV and vaccinia virus clearance during a secondary immune response. These studies have not only allowed us to dissect the PKR-dependent and independent signaling pathways of Type I IFN actions during viral infections, they underscore the essential role for innate immune mechanisms in controlling viral replication upon re-infection, despite the accelerated responses of hyperactive memory CD8^+^ T cells.

## Results

### LCMV control in PKR^−/−^ mice

**Figure 1:**
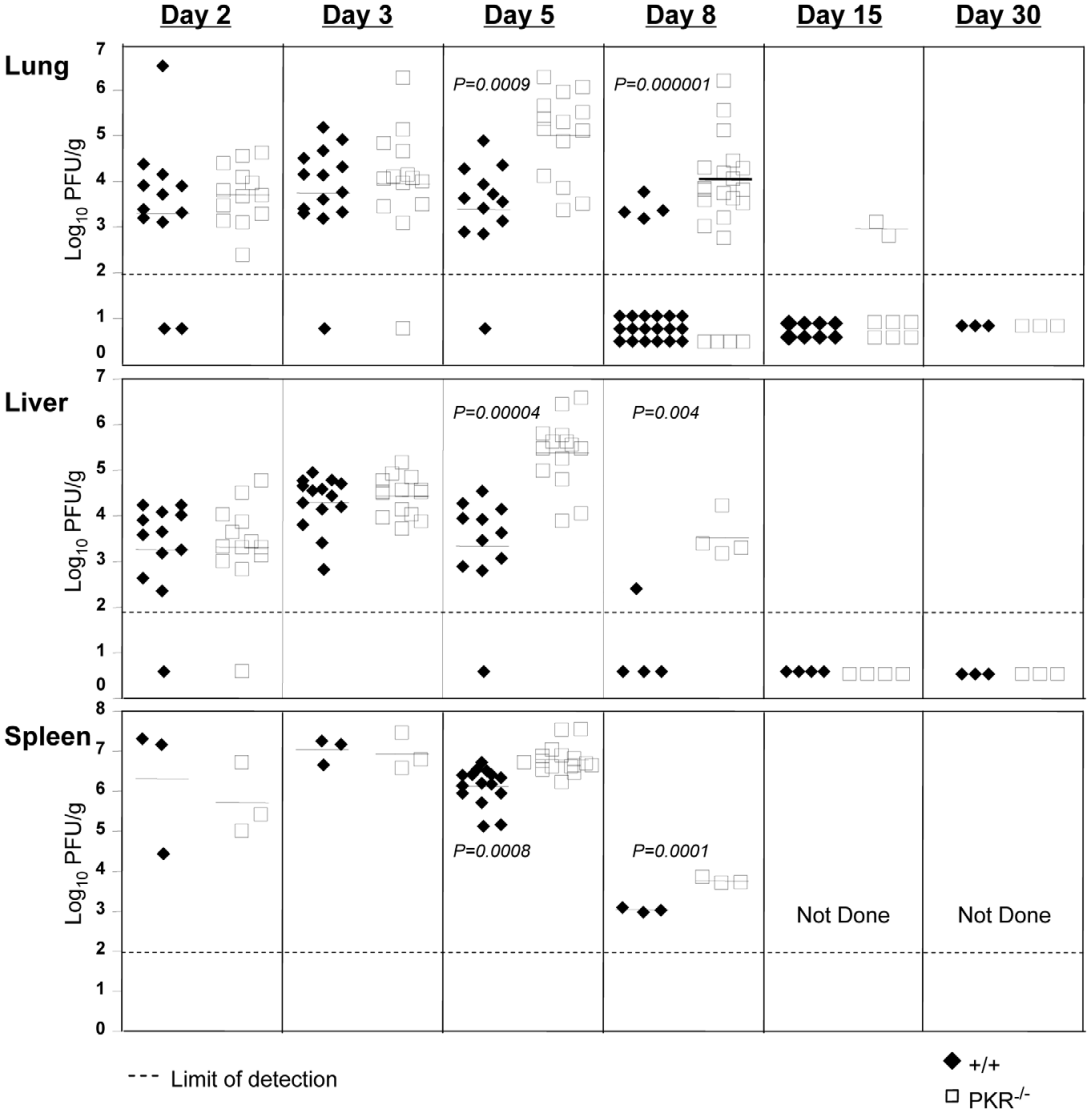
LCMV control in PKR^−/−^ mice. +/+ and PKR^−/−^ mice were infected with LCMV. Lungs, liver, and spleen from +/+ and PKR^−/−^ mice were collected at days 2, 3, 5, 8, 15, and 30 PI and the viral titers were determined by plaque assay. Each symbol (filled symbol is a +/+ mouse and open symbol is a PKR^−/−^ mouse) represents an individual mouse. The horizontal bar is the average titer of the group, and the dotted line is the limit of detection. Data are from 2-6 independent experiments with 3–4 mice/group.

A considerable number of viruses have evolved mechanisms to escape the antiviral effects of PKR, which illustrates its importance in anti-viral innate immunity [15]. However, the effect of PKR deficiency on LCMV control has not been reported. In order to determine whether PKR deficiency affected LCMV replication, wild type (+/+) and PKR-deficient (PKR^−/−^) mice were infected with LCMV, and viral titers in various organs were quantified at days 2, 3, 5, 8, 15 and 30 post-infection (PI). **Figure 1** shows that up to day 3 PI, the LCMV titers in lung, liver, and spleen of PKR^−/−^ mice were comparable to those in +/+ mice. However, between days 3 and 5 PI, differences in viral control emerged between +/+ and PKR^−/−^ mice. Viral titers slightly diminished between day 3 and 5 PI in +/+ mice, whereas, LCMV titers in liver and lungs increased by ∼100-fold in PKR^−/−^ mice during the same interval. Thus, LCMV control at the onset of cellular immunity is compromised in PKR^−/−^ mice. In +/+ mice, a CD8^+^ T cell response is detectable by day 5 PI and peaks on day 8 PI [28]. The emerging virus-specific CD8^+^ T cell response promptly controls viral replication by day 8 to 10 PI [28]. **Figure 1** illustrates that most of the +/+ mice controlled LCMV replication to undetectable levels by day 8 PI. Strikingly, the majority of PKR^−/−^ mice harbored high viral loads in several tissues examined at day 8 PI. However, by day 15 PI, most PKR^−/−^ mice controlled infectious LCMV to levels that were below the limit of detection, with no evidence of viral persistence thereafter. Based on these results, we conclude that PKR plays an important role in controlling LCMV replication in vivo.

### Primary CD8^+^ T cell responses against LCMV in PKR^−/−^ mice

**Figure 2:**
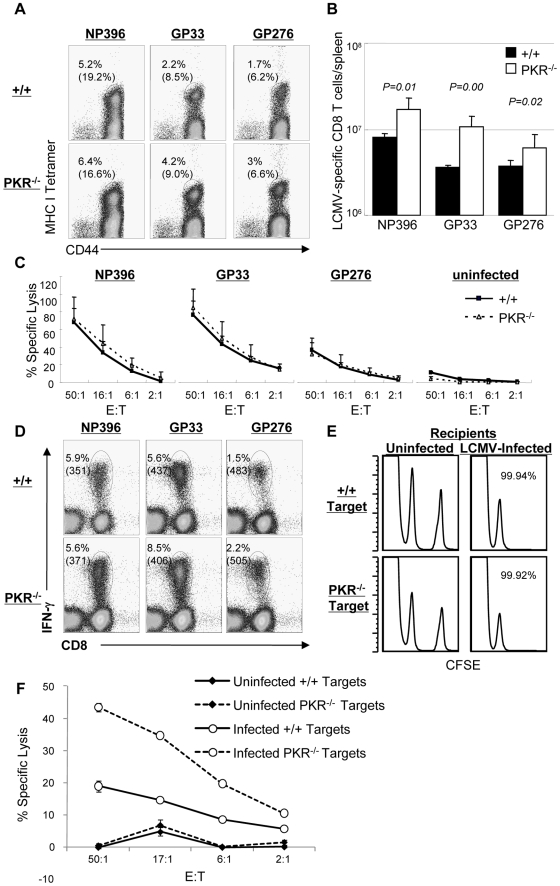
Primary CD8^+^ T cell responses in PKR^−/−^ mice. +/+ and PKR^−/−^ mice were infected intraperitoneally with LCMV, and virus-specific CD8^+^ T cell responses in spleen was assessed at day 8 PI. **A**. Splenocytes were stained with MHC class I tetramers, anti-CD8, and anti-CD44 antibodies. The numbers in the dot plots are the percentages of tetramer positive CD8^+^ T cells amongst splenocytes. The numbers in parenthesis are the percentages of tetramer positive CD8^+^ T cells of total CD8^+^ T cells. **B**. Total numbers of epitope-specific tetramer-binding CD8^+^ T cells in spleen. **C**. Cytotoxic activity of CD8^+^ T cells that are specific to the indicated epitopes was measured by a cytotoxicity assay directly ex vivo. The E:T represents the ratio between splenocytes and peptide pulsed target cells (X axis) **D**. Antigen-triggered IFN-γ production. IFN-γ production by LCMV-specific CD8^+^ T cells was assessed by intracellular cytokine staining (ICCS) in vitro. The numbers on top are the percentages of IFN-γ producing cells among total splenocytes. The number in parenthesis is the mean fluorescent intensity (MFI) of IFN-γ. **E**. The sensitivity of +/+ and PKR^−/−^ target cells to CTL activity was examined by in vivo CTL assay. Splenocytes (target cells) from +/+ and PKR^−/−^ mice were uncoated or coated with GP33 peptide. Uncoated target cells were labeled with a low concentration of CFSE, and GP33-coated target cells were labeled with a high concentration of CFSE. High and low CFSE labeled target cells were mixed 1∶1 and adoptively transferred into naïve (left) or LCMV-infected (day 8 PI) +/+ (right) recipient. Five hours after cell transfer, the presence of donor cells in spleen was analyzed by flow cytometry; the number is the calculated percent specific lysis of adoptively transferred target cells. **F**. Cell-mediated lysis of LCMV-infected BMDCs from +/+ or PKR^−/−^ mice. Primary BMDCs derived from +/+ or PKR^−/−^ mice were infected with LCMV (1.0 MOI) and used as target cells for lysis by effector CD8^+^ T cells from spleen of LCMV-infected C57BL/6 mice (day 8 PI). The E:T represents the ratio between splenocytes (effectors) and LCMV-infected target cells (X axis). Data in this figure are representative of two or more independent experiments with 3–4 mice/group.

Data in **Figure 1** showed that LCMV control was compromised in PKR^−/−^ mice. Here, we determined whether lack of viral control in PKR^−/−^ mice could be linked to the development of an ineffective virus-specific CD8^+^ T cell response. Moreover, we were interested to determine whether Type I IFN-driven CD8^+^ T cell responses [6] to LCMV also required PKR. To this end, +/+ and PKR^−/−^ mice were infected intraperitoneally (i.p.) with LCMV. At day 8 PI, the number of LCMV-specific CD8^+^ T cells in spleen was quantitated by staining with MHC I tetramers (**Figure 2A**). As shown in **Figure 2A and 2B**, the expansion of LCMV-specific CD8^+^ T cells at day 8 PI was significantly greater in PKR^−/−^ mice than in +/+ mice. Next, we examined the role of PKR in regulating the functional attributes of LCMV-specific effector CD8^+^ T cells. As shown in **Figure 2C**, the MHC I-restricted cytotoxic activity of epitope-specific CD8^+^ T cells in PKR^−/−^ mice was comparable to those in +/+ mice. Furthermore, PKR deficiency did not affect antigen-triggered production of IFN-γ (**Figure 2D**) or TNFα (data not shown) by LCMV-specific CD8^+^ T cells. These data suggested that: 1) PKR is not required for elicitation of the CD8^+^ T cell response to LCMV; 2) Lack of viral control in PKR^−/−^ mice cannot be linked to a defect in the anti-viral CD8^+^ T cell response.

**Figure 3:**
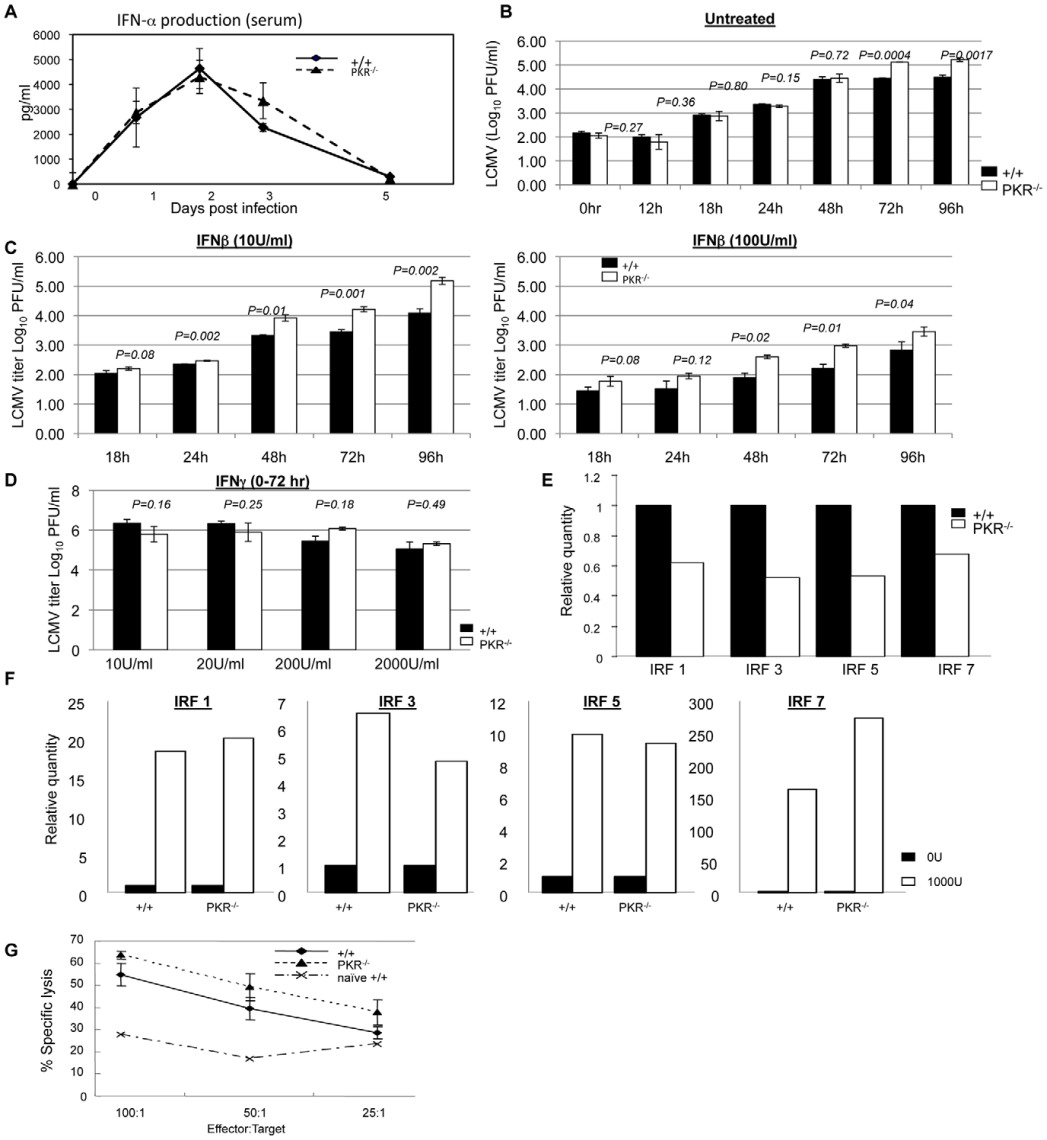
Innate immune responses to LCMV in PKR^−/−^ mice. **A**. +/+ and PKR^−/−^ mice were infected with LCMV and serum samples were collected at days 0, 1, 2, 3, and 5 PI (3–4 mice/group/time point). IFN-α levels in serum were measured using an ELISA kit. **B**. Triplicate cultures of primary bone marrow-derived dendritic cells (BMDCs) from +/+ and PKR^−/−^ mice were infected with LCMV at 0.01 MOI. The supernatants were collected at the indicated time points and viral titers were determined by plaque assay. C. Triplicate cultures of primary BMDCs from +/+ and PKR^−/−^ mice were pretreated with the indicated levels of IFN-β for 20 to 24 hours, then infected with LCMV at 0.01 MOI. The supernatants were collected at the indicated time points and viral titers were determined by plaque assay. **D**. Triplicate cultures of BMDCs from +/+ and PKR^−/−^ mice were left untreated or pretreated with indicated doses of IFN-γ for 20 to 24 hours, then infected with LCMV at 0.01 MOI. The supernatants were collected at 72 hours after infection and viral titers were determined by plaque assay. **E**. Steady state expression levels of IRF-1, IRF-3, IRF-5, and IRF-7 in BMDCs from +/+ or PKR^−/−^ mice. Real-time PCR was used to quantitate mRNA for the indicated IFN-responsive genes. The data are the quantities of mRNAs, relative to expression levels of each gene in cells from +/+ mice. **F**. Expression of IRF-1, IRF-3, IRF-5, and IRF-7 in +/+ and PKR^−/−^ IFN-β-treated BMDCs. Relative quantities of mRNAs were calculated based on their expression levels in untreated BMDCs from respective +/+ or PKR^−/−^ mice. **G**. NK cell activity in the spleens of +/+ and PKR^−/−^ mice (n = 3) that were infected with LCMV three days before was measured by NK cell assay. Data in this figure are representative of at least two independent experiments.

Impaired clearance of LCMV at day 8 PI in PKR^−/−^ mice (**Figure 1**) was surprising, because the virus-specific CD8^+^ T cell response appeared to be unaffected or even stronger in PKR deficient mice. LCMV clearance by CD8^+^ T cells is dependent upon perforin-dependent MHC I-restricted cell-mediated cytotoxicity. It is therefore possible that PKR-deficient target cells may be relatively resistant to cytotoxicity by CD8^+^ T cells, which in turn may impede viral control. To address this issue, we compared the susceptibility of +/+ and PKR^−/−^ splenocytes to cytotoxicity using an in vivo CTL assay. Peptide-pulsed splenocytes from +/+ or PKR^−/−^ mice were adoptively transferred into LCMV-infected C57BL/6 mice, and in vivo lysis of the donor cells was assessed by flow cytometry. As shown in **Figure 2E**, LCMV-specific effector CD8^+^ T cells lysed peptide-coated PKR^−/−^ and PKR^+/+^ target cells similarly in vivo. It is also possible that PKR might regulate the sensitivity of LCMV-infected cells to lysis by effector CD8^+^ T cells. To address this issue, we tested the susceptibility of LCMV-infected primary bone marrow-derived dendritic cells (BMDCs) from +/+ and PKR^−/−^ mice to effector CD8^+^ T cell-mediated cytotoxicity in vitro. Data in **Figure 2F** show that LCMV-specific effector CD8^+^ T cells lysed LCMV-infected +/+ and PKR^−/−^ BMDCs. Surprisingly, lysis of PKR^−/−^ BMDCs was higher, as compared to +/+ BMDCs (**Figure 2F**), which might be linked to enhanced infection of PKR^−/−^ cells by LCMV (see **Figure 3B**). Taken together, we conclude that PKR deficiency does not appear to affect sensitivity of peptide-coated or LCMV-infected target cells to lysis by CD8^+^ T cells.

Data in **Figure 1** show that LCMV titers in PKR^−/−^ mice but not +/+ mice increased between days 3 and 5 PI, which coincides with the initiation of the anti-LCMV T cell response. First, we examined whether such an increase in viral titers occurred in RAG1-deficient (RAG1^−/−^) mice that lacks both B and T cells. As shown in **Figure S1**, interestingly, viral titers increased between days 3 and 5 PI only in RAG1^−/−^ mice but not in control +/+ mice. These data indicated that increase in LCMV titers in PKR^−/−^ mice between day 3 and 5 PI might be linked to delayed induction of CD8^+^ T cell response. To address this issue, we quantitated CD8^+^ T cell response of +/+ and PKR^−/−^ mice to LCMV at day 5 PI. Data in **Figure S2** show that LCMV-specific CD8^+^ T cell response in PKR^−/−^ mice at day 5 PI was higher than in +/+ mice. Therefore, it is unlikely that delayed development of CD8^+^ T cell response contributed to ineffective control of LCMV in PKR^−/−^ mice. However, a possibility still exists that CD8^+^ T cells might require PKR activity in infected cells to suppress cellular viral replication. Further investigations will be required to determine whether viral suppression by LCMV-specific CD8^+^ T cells might occur less efficiently in the absence of PKR activity in virally infected cells, at least early in the infection.

### Innate immune responses in PKR^−/−^ mice

Data in **Figure 1** **and** **2** showed that PKR^−/−^ mice exhibit less effective control of LCMV infection, despite the elicitation of a potent CD8^+^ T cell response. These findings suggested a role for PKR in mediating at least some aspects of the innate response to LCMV. It is well established that Type I IFNs play a critical role in controlling LCMV replication early in the infection [6], [25], [26], and it has been reported that both induction of Type I IFNs and some of the antiviral effects of Type I IFN receptor signaling are mediated via PKR [29]. Therefore, less effective viral control might be due to lower production of Type I IFNs and/or a reduction in the antiviral effects induced by Type I IFN receptor signaling. To explore these possibilities, we examined whether IFN-α production is affected by PKR deficiency during a LCMV infection. As shown in **Figure 3A**, following an acute LCMV infection, there was a substantial induction of IFN-α in the serum of both +/+ and PKR^−/−^ mice. The level of IFN-α peaked at 48 hours PI and tapered off thereafter in +/+ and PKR^−/−^ mice. Importantly, at all time points serum levels of IFN-α in PKR^−/−^ mice were similar to those in +/+ mice. Thus, PKR deficiency did not affect production of IFN-α during an acute LCMV infection.

Next, we determined whether lack of innate cellular resistance to virus lead to enhanced LCMV replication in PKR-deficient cells. BMDCs from +/+ and PKR^−/−^ mice were infected with LCMV and viral titers were quantitated at different time points after infection. The kinetics of viral replication in PKR^−/−^ cells was similar to +/+ cells until 48 hrs. After 48 hours, however, viral production by PKR^−/−^ BMDCs was reproducibly higher, as compared to +/+ DCs (**Figure 3B**) which suggested that LCMV replication is enhanced in PKR^−/−^ cells. Since the Type I IFN-induced antiviral state is dependent at least in part on PKR, it is possible that Type I IFN signaling-induced suppression of LCMV replication is impaired in PKR^−/−^ mice. Therefore, we determined whether PKR is required for Type I IFNs to effectively suppress LCMV replication by comparing the viral titers in +/+ and PKR^−/−^ cells pretreated with IFN-β in vitro. Primary BMDCs from +/+ and PKR^−/−^ mice were incubated for 20 to 24 hours with two different concentrations of IFN-β prior to infection with LCMV; viral titers were determined at different times after LCMV infection (**Figure 3C**). Pretreatment of cells with IFN-β reduced viral titers in cultures of +/+ and PKR^−/−^ BMDCs in a dose-dependent fashion. We compared the effects of IFN-β on viral titers in +/+ and PKR^−/−^ BMDCs by calculating percent reduction in viral titers induced by IFN-β, relative to titers in untreated cells (**Figure 3C**); note that linear and not log_10_ virus titers were used for this calculation. **Figure S3** illustrates the IFN-β treatment -induced reduction in LCMV titers in +/+ and PKR^−/−^ BMDCs from two independent experiments. In both experiments, IFN-β treatment led to a reduction in LCMV titers in cultures of both +/+ and PKR^−/−^ BMDCs. At 24, 48, and 72 hours after IFN-β treatment (10 U/ml), there were variable and modest differences in viral titer reduction (5–20%) between +/+ and PKR^−/−^ BMDCs (**Figure S3**). However, at 96 hours after IFN-β treatment, in both experiments, IFN-β-induced reduction in viral titers in PKR^−/−^ BMDCs was significantly (*P*<0.05) lower, as compared to those in +/+ BMDCs. Viral titer reduction by IFN-β treatment at a higher concentration (100 U/ml) was comparable in +/+ and PKR^−/−^ BMDCs. Thus, the anti-viral effects of IFN-β are largely intact, but might be a little short-lived in the absence of PKR (**Figure S3**). Additionally, the cell surface expression of Type I IFN receptor was unaffected by PKR deficiency (**Figure S4**). Treatment of +/+ BMDCs with IFN-γ also led to a modest reduction in virus titers with the effect much less pronounced for BMDCs from PKR^−/−^ mice (**Figure 3D**). Taken together, these data suggested that: 1) PKR plays a role in suppressing LCMV replication in infected cells; and 2) the antiviral effects of Type I IFNs are largely intact, but might be less sustained in PKR-deficient cells.

To further characterize the role of PKR in regulation of LCMV replication, we quantitated the expression of IFN-regulated genes with or without treatment with IFN-β. Data in **Figure 3E** show that basal expression level of IFN-regulatory factors (IRFs) was 30 to 50% lower in BMDCs from PKR^−/−^ mice than in +/+ mice. This might explain the enhanced LCMV replication in PKR^−/−^ BMDCs in vitro (**Figure 3B**). Treatment with IFN-β for 6 hours led to comparable upregulation of transcripts for IRF1, IRF3, IRF5, and IRF7 genes in +/+ and PKR^−/−^ cells, as compared to unstimulated cells (**Figure 3F**). Next we compared the levels of the transcripts for these IFN-regulated genes in IFN-β-treated +/+ and PKR^−/−^ cells. The transcript levels for IRF1, IRF3, and IRF5, but not IRF-7 was ∼50% lower in IFN-β-treated cells from PKR^−/−^ mice compared to IFN-β-treated BMDCs from +/+ mice (not shown). These findings show that PKR plays a positive regulatory role in governing the cellular levels of ISGs in steady state or even upon exposure to IFN-β.

Activation of natural killer cells (NK cells) constitutes an important facet of innate immunity to virus infection. NK cells typically are activated by Type I IFNs from day 2 to 5 after infection; peak NK cell activity is detectable at day 3 in LCMV infection. Although NK cells are not required to clear LCMV [30], it is possible that lower NK cell activity might facilitate LCMV proliferation in PKR^−/−^ mice. Here we determined whether PKR is required for NK cells activation in vivo. **Figure 3G** show that NK cells from both +/+ and PKR^−/−^ mice lysed target cells efficiently. Thus, PKR is not required for NK cell activation during an acute LCMV infection.

### Secondary CD8^+^ T cells responses and protective immunity to LCMV in PKR^−/−^ mice

**Figure 4:**
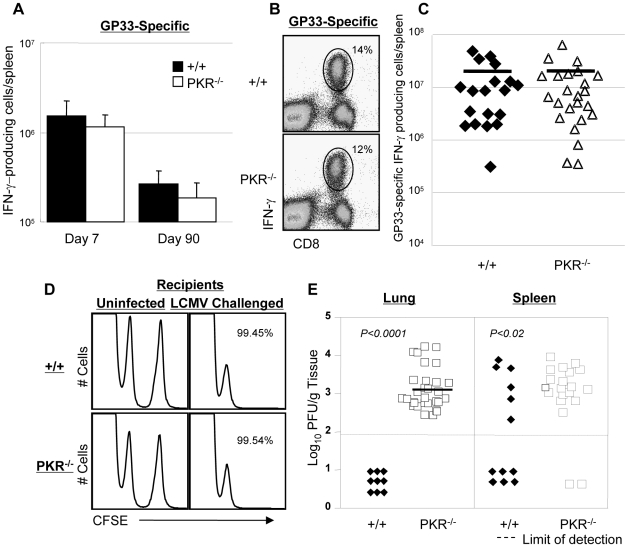
Primary and secondary CD8^+^ T cell responses after *Listeria monocytogenes* immunization. **A**. +/+ and PKR^−/−^ mice were infected with rLM-GP33. At 7 and 90 days after infection, the numbers of GP33-specific IFN-γ-producing CD8^+^ cells were quantitated by intracellular cytokine staining. **B and C**. At day 90 PI, LM-GP-immune +/+ and PKR^−/−^ mice were challenged with LCMV. Five days after challenge, the number of GP33-specific IFN-γ-producing CD8^+^ T cells was assessed by intracellular cytokine staining. Dot plots from representative mice (**B**) and the absolute numbers of all mice (**C**) are shown. The horizontal bar in **C** is the average for each group. **D**. CTL activity of GP33-specific effector CD8^+^ T cells in spleen of LCMV-challenged +/+ and PKR^−/−^ mice was measured by in vivo CTL assay at day 5. GP33-coated target cells (CFSE^high^) or uncoated target cells (CFSE^low^) were transferred into uninfected or LCMV-challenged (Chal-D5) +/+ or PKR^−/−^ mice. The number is the calculated percent specific lysis of adoptively transferred target cells, and representative of data from 3–4 mice/group. **E**. Viral load in lung and spleen of LCMV-challenged +/+ or PKR^−/−^ mice at day 5. Each symbol represents an individual mouse; filled symbols are +/+ and open symbols are PKR^−/−^ mice. Data in this figure are representative of or derived from 2–8 independent experiments with 3–4 mice/group.

Data in **Figure 1** clearly illustrated that PKR is required for normal viral clearance during a primary LCMV infection. Although it is well established that innate immune mechanisms are important in viral control during a primary infection, it is unknown whether innate effectors like PKR are required for efficient viral clearance during a secondary CD8^+^ T cell response. Therefore, we investigated the effect of PKR deficiency on memory CD8^+^ T cell-dependent LCMV control during a secondary T cell response. Groups of +/+ and PKR^−/−^ mice were immunized with recombinant *Listeria monocytogenes* that expressed the GP33 epitope of LCMV (rLM-GP33). Both +/+ and PKR^−/−^ mice mounted a strong primary CD8^+^ T cell response to rLM-GP33; the number of GP33-specific CD8^+^ T cells at the peak of the primary response (day 7 PI) and memory (day 90 PI) were similar in +/+ and PKR^−/−^ mice (**Figure 4A**). Thus, PKR deficiency did not affect the development of CD8^+^ T cell memory following immunization with rLM-GP33 in mice. Next, we examined the importance of PKR in LCMV control during a secondary CD8^+^ T cell response. Groups of +/+ and PKR^−/−^ mice were immunized with rLM-GP33, and then challenged with LCMV. Five days after LCMV challenge, secondary expansion of GP33-specific CD8^+^ T cells was assessed in the spleens of +/+ and PKR^−/−^ mice. **Figure 4B** shows that GP33-specific CD8^+^ T cells expanded to comparable levels in both +/+ and PKR^−/−^ mice. The absolute numbers of expanded CD8^+^ T cells were consistent between +/+ and PKR^−/−^ mice (**Figure 4C**). To assess whether GP33-specific memory CD8^+^ T cells differentiated into effectors in LCMV-challenged +/+ and PKR^−/−^ mice, we performed an in vivo CTL assay. The in vivo CTL activity in the spleens of PKR^−/−^ mice was undistinguishable from that in +/+ mice (**Figure 4D**). Consistent with potent secondary expansion of CD8^+^ T cells and effector activity, +/+ mice controlled LCMV effectively in both lung and spleen. In striking contrast, PKR^−/−^ mice exhibited poor LCMV control in lung and spleen despite strong secondary CD8^+^ T cell responses (**Figure 4E**). Taken together, the data in **Figures 1** and **4** show that PKR is required for viral control during primary and secondary CD8^+^ T cell response to LCMV infection. Please note that PKR^−/−^ mice do control LCMV during a primary infection, albeit after a delay of ∼7 days (**Figure 1**). Therefore, it is expected that potent secondary GP33-specific CD8^+^ T cell responses in LCMV-challenged rLM-GP33-immune mice would be able to control LCMV without the establishment of viral persistence.

### Viral control by TCR transgenic memory CD8^+^ T cells in PKR deficient mice

**Figure 5:**
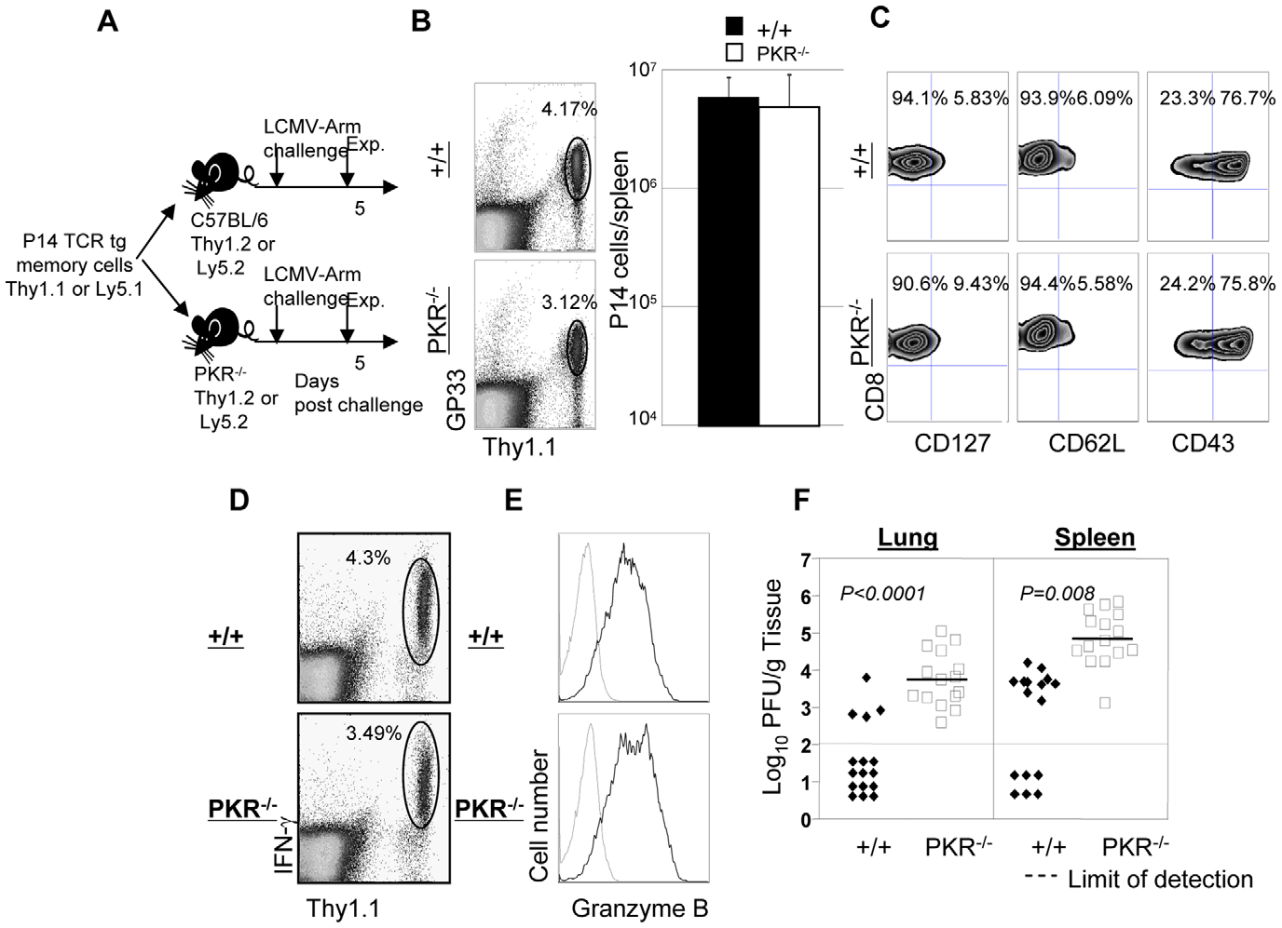
Secondary expansion of TCR tg memory CD8^+^ T cells in +/+ and PKR^−/−^ mice. **A**. Experimental design. Thy1.1^+ve^ P14 memory CD8^+^ T cells (generated as described in Materials and Methods), were adoptively transferred into congenic Thy1.2/+/+, or Thy1.2/PKR^−/−^ mice. Following cell transfer, mice were challenged with LCMV, and secondary expansion of donor P14 CD8^+^ T cells was assessed in spleen at day 5 after challenge. **B and C**. Splenocytes from +/+ or PKR^−/−^ were stained with D^b^/GP33 MHC I tetramer and anti-Thy1.1; dot plots are gated on total splenocytes, and the numbers are the percentages of P14 CD8^+^ T cells amongst splenocytes. The total number of P14 cells (right panel in **B**) is calculated from 5 mice/group. **C**. Cell surface phenotype of donor P14 CD8^+^ T cells in LCMV-challenged +/+ and PKR^−/−^ mice; FACS plots are gated on tetramer-binding P14 CD8^+^ T cells. **D**. IFN-γ production by donor P14 cells was quantitated by intracellular cytokine staining; dot plots are gated on total splenocytes. **E**. Splenocytes from LCMV-challenged +/+ or PKR^−/−^ mice were stained for cell surface Thy1.1/CD8 and intracellular granzyme B. FACS histograms are gated on P14 CD8^+^ T cells; dotted and solid lines represent staining with isotype control and anti-granzyme B antibodies respectively. **F**. Viral titers in lung and spleen of LCMV-challenged +/+ or PKR^−/−^ mice were determined by plaque assay. Each symbol represents an individual mouse. Results are representative of or derived from 4 independent experiments with 3 to 5 mice per group.

Data in **Figure 4** show that PKR^−/−^ mice immunized with rLM-GP33 provided poor protective immunity against LCMV, despite robust secondary CD8^+^ CTL responses. One caveat to these experiments is that the total number and quality of LCMV-specific memory CD8^+^ T cells (prior to LCMV challenge) in PKR^−/−^ mice could be lower than in +/+ mice. Alternatively, it is possible that memory CD8^+^ T cells generated in PKR^−/−^ mice might possess poor protective abilities. Furthermore, viral clearance might be impaired in PKR^−/−^ mice regardless of the quality and the magnitude of the secondary CD8^+^ T cell response, due to deficiency of other factors involved in LCMV control. Here we tested the hypothesis that PKR expression in non CD8^+^ T cells is required for memory CD8^+^ T cell-dependent control of LCMV. To test this hypothesis, we generated wild type monoclonal TCR transgenic (tg) P14 LCMV-specific memory CD8^+^ T cells in vivo in a PKR-sufficient environment. Thy1.1 positive wild type GP33-specific TCR tg P14 memory CD8^+^ T cells were adoptively transferred into cohorts of +/+ or PKR^−/−^ mice, which were subsequently challenged with LCMV (**Figure 5A**). Please note that adoptively transferred P14 CD8^+^ T cells are Thy1.1 positive while T cells in the recipients are Thy1.2 positive. Five days after challenge, the secondary expansion of donor P14/Thy1.1 memory CD8^+^ T cells and viral control were assessed in +/+ and PKR^−/−^ mice. Donor P14 memory CD8^+^ T cells expanded in both +/+ and PKR^−/−^ recipient mice, and the total number of P14 memory CD8^+^ T cells in spleen of PKR^−/−^ mice was comparable to those in +/+ mice (**Figure 5B**). Furthermore, secondary P14 effectors in +/+ and PKR^−/−^ recipients showed downregulation of CD127 and CD62L, and upregulation of CD43 expression (**Figure 5C**). All donor P14 CD8^+^ T cells in LCMV-challenged +/+ or PKR^−/−^ recipients were functional, readily produced IFN-γ upon ex vivo antigenic stimulation (**Figure 5D**), and also expressed high levels of granzyme B (**Figure 5E**). In summary, the adoptively transferred P14 CD8^+^ T cells in PKR^−/−^ mice were comparable to those in +/+ mice based on number, cell surface phenotype, and function. While 75% of +/+ mice cleared LCMV to levels below the limit of detection, all PKR^−/−^ mice uniformly contained high levels of virus in the lung. The spleens of PKR^−/−^ mice contained >100 fold greater levels of infectious LCMV than in +/+ mice (**Figure 5F**). Thus, LCMV control by wild type P14 memory CD8^+^ T cells was less efficient in a PKR-deficient environment as compared to a PKR-sufficient environment. However, as in a primary infection (**Figure 1**), LCMV-challenged PKR^−/−^ mice would be expected to achieve viral clearance, albeit with a delay of ∼1 week.

**Figure 6:**
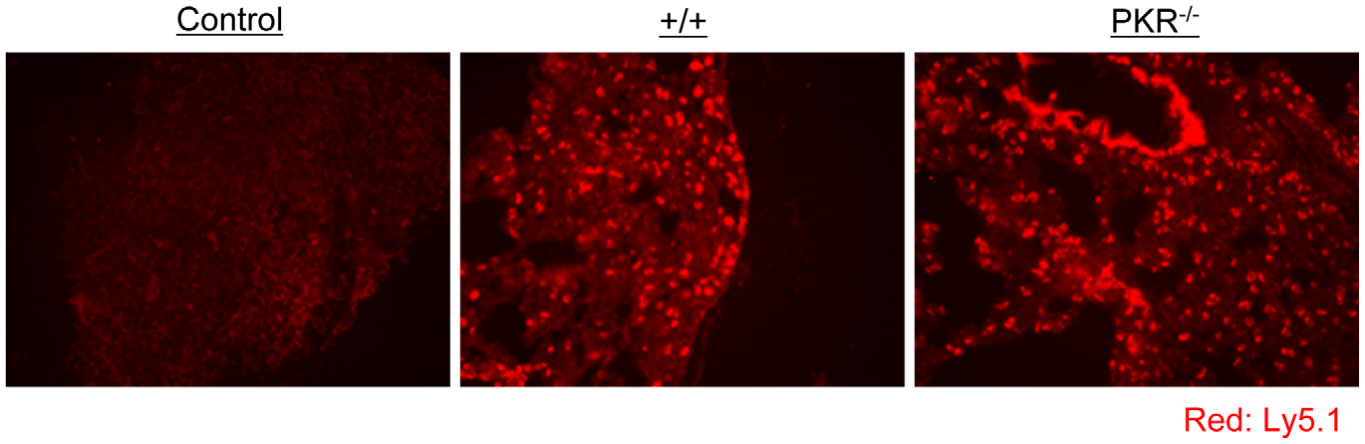
Trafficking of TCR tg P14 CD8^+^ T cells into infected lungs in +/+ and PKR^−/−^ mice. As in Figure 5, Ly5.1/P14 TCR tg memory CD8^+^ T cells were adoptively transferred into +/+ or PKR^−/−^ mice, which were subsequently challenged with LCMV. Five days after challenge, trafficking of Ly5.1/P14 CD8^+^ T cells into lungs was visualized by immunofluorescent staining with Alexa 568-conjugated anti-Ly5.1 antibodies (Red). Lung section from a +/+ mouse that did not receive Ly5.1/P14 CD8^+^ T cells is shown as a negative control. Multiple sections from each mouse were analyzed, and results in this figure are representative of data from 3 to 4 mice per group.

It has been reported that PKR might play a role in inducing the expression of adhesion molecules, such as vascular cell adhesion molecule (VCAM)-1, which controls immune cell migration [31]. To examine the possibility that P14/Ly5.1 T cell trafficking to the infected sites might be defective in PKR deficient mice, lung sections from P14 cell transferred +/+ and PKR^−/−^ mice were stained with anti-Ly5.1 antibody. In **Figure 6**, TCR tg donor Ly5.1 positive P14 cells were detected in lungs of recipient +/+ and PKR^−/−^ mice, but not in control mice that did not receive P14 cells. The anatomical distribution and number of Ly5.1/P14 CD8^+^ T cells in the lungs of PKR^−/−^ mice was similar to those in +/+ mice. These data suggested that PKR deficiency might not affect trafficking of effector CD8^+^ T cells to the site of infection.

### Viral control by polyclonal PKR-deficient memory CD8^+^ T cells

**Figure 7:**
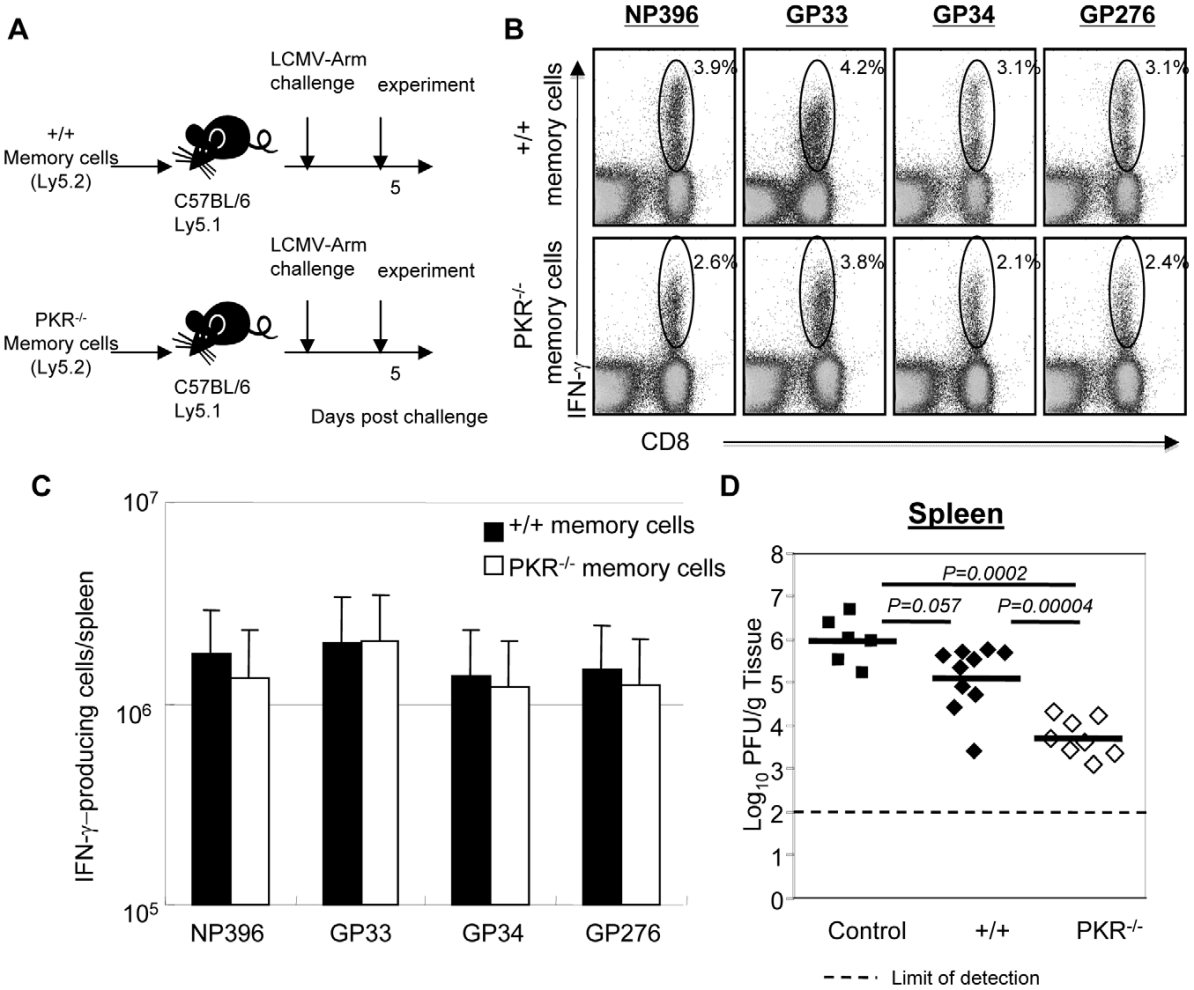
Secondary expansion of +/+ or PKR^−/−^ memory CD8^+^ T cells in Ly5.1/+/+ mice. **A**. Experimental design. Ly5.2/+/+ or Ly5.2/PKR^−/−^ mice were infected with LCMV. At 90 days after LCMV infection, CD8^+^ T cells were purified from spleens of LCMV-immune +/+ or PKR^−/−^ mice. Total CD8^+^ T cells containing equal number of LCMV-specific memory CD8^+^ T cells (NP396+GP33+GP276-specific) were adoptively transferred into congenic C57BL/6/Ly5.1+/+ mice. The recipients were challenged with LCMV and the secondary expansion of donor LCMV-specific memory CD8^+^ T cells was assessed 5 days later. **B and C**. Antigen-triggered IFN-γ production by donor Ly5.2^+^ CD8^+^ T cells was quantitated by intracellular cytokine staining. **B**. The numbers in the dot plots are the percentage of IFN-γ-producing CD8^+^ T cells among total splenocytes. **C**. Absolute number of donor antigen-specific CD8^+^ T cells in the recipient. **D**. The viral titer in spleen of LCMV-challenged mice that were recipients of +/+ or PKR^−/−^ memory CD8^+^ T cells was quantitated by plaque assay; viral titer in mice that did not receive memory CD8^+^ T cells are shown as controls. Each symbol indicates an individual mouse and the horizontal bar is the average of the group. Results are representative of two independent experiments with 4 to 6 mice per group.

Data in **Figure 5F** illustrates that P14 TCR tg memory CD8^+^ T cells that express PKR controlled LCMV in +/+ and not in PKR^−/−^ mice. This finding suggested that PKR in non CD8^+^ T cells contributes to LCMV clearance. Here, we investigated whether PKR expression in CD8^+^ T cells regulates viral clearance by LCMV-specific memory CD8^+^ T cells. Groups of +/+ and PKR^−/−^ mice were infected with LCMV. At day 90 PI, we quantitated the number of LCMV-specific memory CD8^+^ T cells in spleens of LCMV-immune +/+ and PKR^−/−^ mice by using MHC I tetramers and intracellular cytokine staining. These studies showed that the total number of CD8^+^ T cells specific to the three dominant epitopes (NP396, GP33, and GP276) was ∼2-fold higher in PKR^−/−^ mice than in +/+ mice (data not shown). We also compared the relative proportions of central (CD62L^Hi^) and effector (CD62L^Lo^) LCMV-specific memory CD8^+^ T cells between LCMV-immune +/+ and PKR^−/−^ mice. The percentages of effector memory cells amongst +/+ and PKR^−/−^ memory CD8^+^ T cells ranged between 20–30% and 50–70% respectively (**Figure S5**). The increase in the relative proportions of effector memory CD8^+^ T cells is likely linked to delayed viral clearance in PKR^−/−^ mice [32]. CD8^+^ T cells were purified from the spleens of LCMV-immune +/+ and PKR^−/−^ mice, and the numbers of memory CD8^+^ T cells that are specific to the three dominant epitopes were normalized by tetramer staining. Purified CD8^+^ T cells (containing equal number of LCMV-specific memory CD8^+^ T cells) from +/+ or PKR^−/−^ mice (Ly5.2) were adoptively transferred into congenic Ly5.1/B6 mice, which were subsequently challenged with LCMV (**Figure 7A**). At 5 days after challenge, we assessed the secondary expansion of donor CD8^+^ T cells and viral load in tissues. As shown in **Figure 7B and 7C**, the secondary expansion of donor PKR^−/−^ mice memory CD8^+^ T cells was comparable to +/+ memory CD8^+^ T cells. Viral load in recipient mice was compared to control mice that did not receive memory CD8^+^ T cells. As expected, control mice that were not recipients of memory CD8^+^ T cells harbored high levels of virus in the spleen. Although protection was not complete, +/+ memory CD8^+^ T cells and PKR-deficient memory CD8^+^ T cells reduced the viral load by 10 to 100-fold, respectively, compared to control mice (**Figure 7D**). Importantly, viral control by PKR^−/−^ memory CD8^+^ T cells was superior to +/+ memory CD8^+^ T cells, which suggested that PKR expression in memory CD8^+^ T cells is not required for LCMV control. The mechanisms underlying the enhanced protection by PKR-deficient memory CD8 T cells need further investigation. There is increasing evidence that effector memory CD8^+^ T cells confer better protection than the central memory CD8^+^ T cells [33], [34], [35], [36]. Therefore, we speculate that larger number of effector memory CD8 T cells present amongst the adoptively transferred memory PKR^−/−^ memory CD8^+^ T cells led to enhanced LCMV control in recipient mice. Taken together, the data in **Figures 5** and **7** show that PKR expression in the infected cells, but not in CD8^+^ T cells, is important for effective viral control.

### Secondary responses and protection by memory CD8^+^ T cells in IFNAR^−/−^ mice

**Figure 8:**
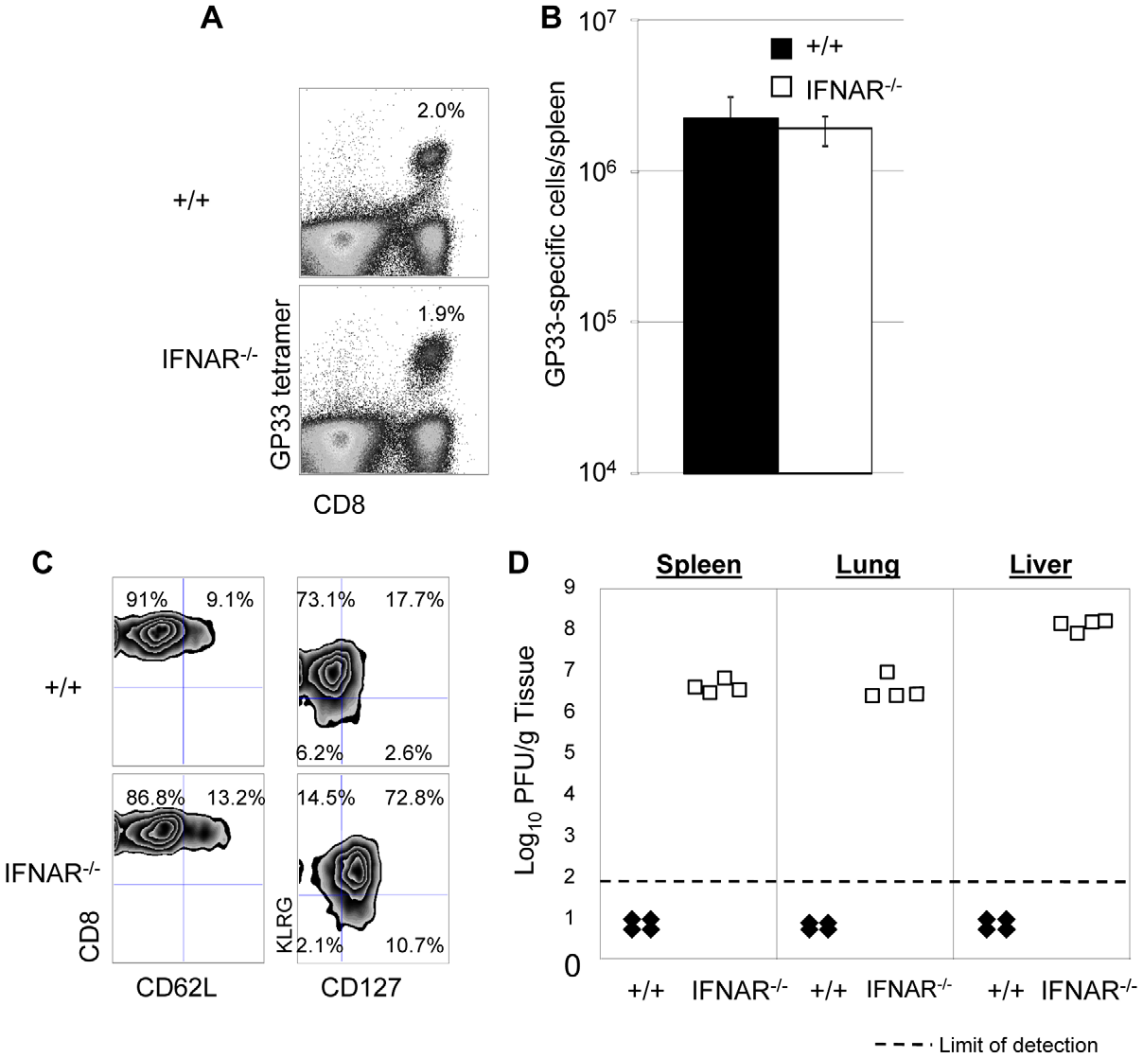
Secondary expansion of GP33-specific CD8^+^ T cells and viral control in +/+ and IFNAR^−/−^ mice. +/+ or IFNAR^−/−^ mice were immunized with rLM-GP33. Sixty days after rLM-GP33 infection, mice were challenged with LCMV and GP33-specific CD8^+^ T cell responses were assessed in spleen five days later. **A**. The dot plots show expansion of GP33-specific CD8^+^ T cells in +/+ and IFNAR^−/−^ mice; the numbers are the percentages of GP33-specific CD8^+^ cells among total splenocytes. **B** shows total number of GP33-specific CD8^+^ T cells in spleen of LCMV-challenged +/+ and IFNRA^−/−^ mice. **C**. Cell surface phenotype of GP33-specific CD8^+^ T cells. Splenocytes from +/+ or IFNAR^−/−^ mice were stained with antibodies against CD62L, CD127, and KLRG-1 in conjunction with anti-CD8 and D^b^/GP33 MHC I tetramers. Dot plots are gated on tetramer-binding CD8^+^ T cells, and the numbers are the percentages of CD62L^hi/low^, CD127^hi/low^, and KLRG-1^hi/low^ cells among tetramer-binding CD8^+^ T cells. **D**. Viral titers of spleen, lung, and liver from LCMV-challenged +/+ or IFNAR^−/−^ mice were quantitated by plaque assay; each symbol indicates viral titer of an individual mouse. Results are representative of two independent experiments, with 3 to 4 mice per group.

The results presented thus far (**Figures 1** **,** **4** **, and** **5**) demonstrated that PKR is required for LCMV clearance during primary and secondary infection in mice. PKR activity is induced by Type I IFNs, and some of the antiviral activity of the Type I IFNs could be PKR dependent (**Figure 3C**). We, therefore, hypothesized that Type I IFN signaling is required for protection against secondary LCMV infection, which could be, at least in part, PKR dependent. In order to test this hypothesis, we took two approaches: In the first set of experiments, we immunized +/+ or Type I IFN receptor-deficient (IFNAR^−/−^) mice with rLM-GP33, which were challenged with LCMV (described in **Figure 4**). GP33-specific memory CD8^+^ T cells generated by immunization with rLM-GP33 expanded similarly in +/+ or IFNAR^−/−^ mice following LCMV challenge (**Figure 8**). **Figures 8A and 8B** show secondary expansion of GP33-specific CD8^+^ T cells. The expressions of CD62L (the lymphocyte homing receptor) and CD127 (the IL-7 receptor) were low, but the expression of KLRG-1 (the NK cell receptor) was high on effector cells in +/+ mice (**Figure 8C**). Notably, CD127 expression on effector CD8^+^ cells in IFNAR^−/−^ mice was markedly higher (**Figure 8C**), as compared to those in +/+ mice. Despite the comparable expansion of GP33-specific T cells in both groups of mice, viral control by GP33-specific effector cells in IFNAR^−/−^ mice was severely impaired (**Figure 8D**).

**Figure 9:**
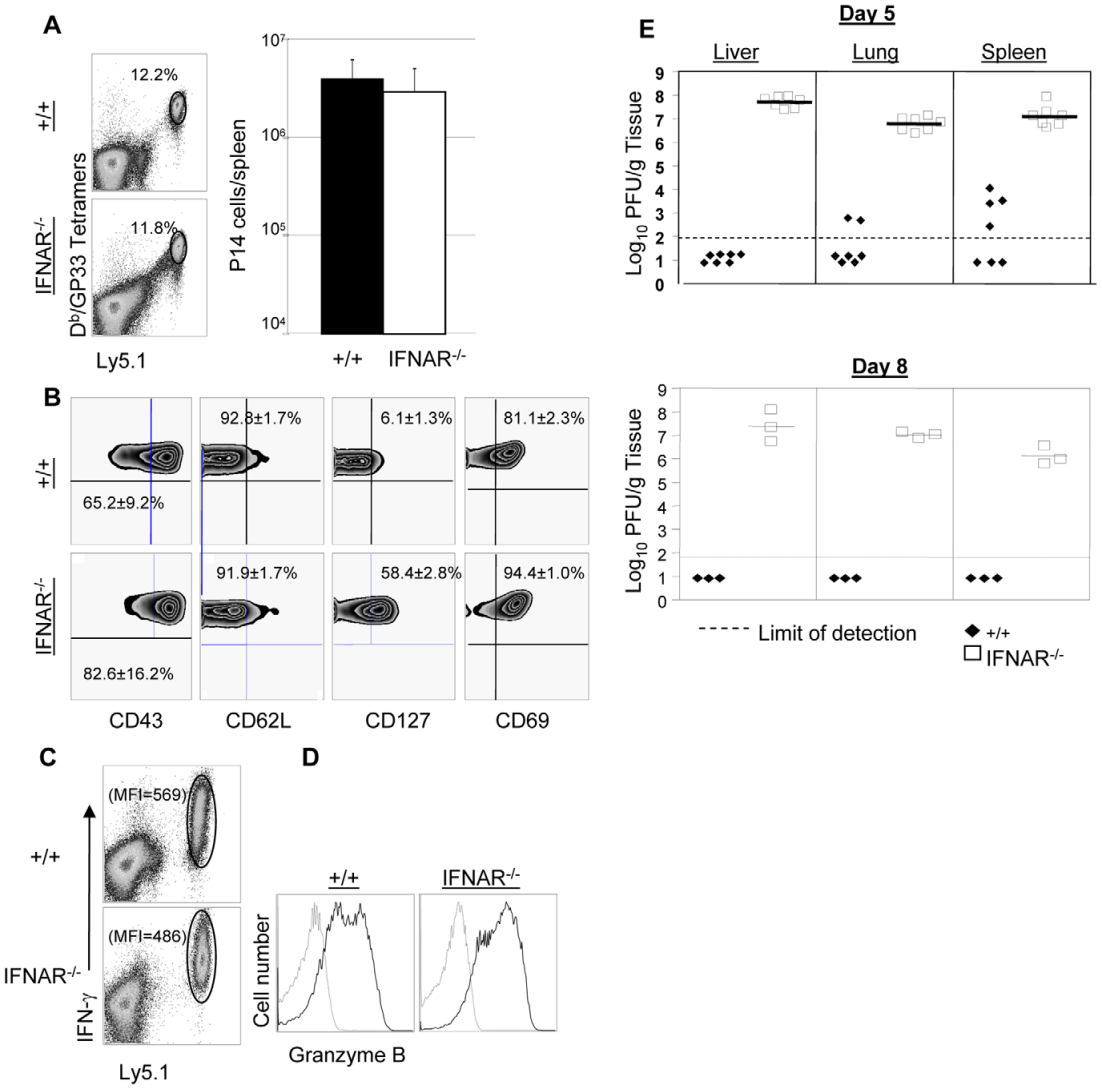
Expansion of TCR tg P14 memory CD8^+^ T cells and viral control in IFNAR^−/−^ mice. As in Figure 5A, Ly5.1/P14 memory CD8^+^ T cells (generated as described in Materials and Methods), were adoptively transferred into congenic Ly5.2/+/+, or Ly5.2/IFNAR^−/−^ mice; recipient mice were challenged with LCMV and CD8^+^ T cell responses were assessed five days later. **A**. Secondary expansion of donor P14 memory CD8^+^ T cells. Dot plots are gated on total splenocytes, and the numbers are percentages of donor Ly5.1 CD8^+^ T cells among splenocytes; the accompanying bar graph (right panel) shows total number of P14 CD8^+^ T cells in spleen of +/+ and IFNRA^−/−^ mice. **B**. Cell surface phenotype of donor P14 CD8^+^ T cells. Splenocytes from +/+ or IFNAR^−/−^ recipients were stained with antibodies against CD43, CD62L, CD127, and CD69 in conjunction with anti-Ly5.1, anti-CD8, and D^b^/GP33 tetramers. FACS plots are gated on tetramer-binding Ly5.1^+ve^ cells, and the numbers are the percentages of CD43^hi^, CD62L^lo^, CD127^hi^, and CD69^hi^ cells among tetramer-binding P14 CD8^+^ T cells. **C**. IFN-γ production by P14 cells from +/+ or IFNAR^−/−^ mice were quantitated by intracellular cytokine staining. Note that all Ly5.1^+ve^ P14 CD8^+^ T cells from +/+ or IFNRA^−/−^ mice produced IFN-γ; the numbers are the MFI for IFN-γ staining. **D**. Granzyme B expression in P14 cells from +/+ or IFNAR^−/−^ mice. Splenocytes were stained with anti-Ly5.1, anti-CD8, D^b^/GP33 tetramers, and anti-granzyme B; FACS histograms are gated on Ly5.1^+ve^ tetramer-binding CD8^+^ T cells; dotted and solid lines show staining with isotype control and anti-granzyme B antibodies respectively. **E**. Viral titers of liver, lung, and spleen from +/+ or IFNAR^−/−^ mice were quantitated by plaque assay; each symbol represents viral titer in an individual mouse. Results are representative of two independent experiments, with 3 to 4 mice per group.

In a second series of experiments, we adoptively transferred P14/Ly5.1 memory CD8^+^ T cells into +/+ or IFNAR^−/−^ mice, which were subsequently challenged with LCMV. The secondary expansion of donor P14/Ly5.1 CD8^+^ T cells and viral control was assessed as described above. **Figure 9A** shows that secondary expansion of P14/Ly5.1 CD8^+^ T cells was comparable in +/+ and IFNAR^−/−^ mice. The cell surface expression of CD43, CD62L, and CD69 in +/+ recipients was similar to those in IFNAR^−/−^ recipients. Interestingly, CD127 expression on P14 CD8^+^ T cells in IFNAR^−/−^ mice was substantially higher than in +/+ mice (**Figure 9B**), which is consistent with the result from rLM-GP33 immunization model. The IFN-γ-producing ability and granzyme B content of P14 CD8^+^ T cells in IFNAR^−/−^ mice was comparable to +/+ mice (**Figures 9C, D**). Strikingly, despite normal secondary CD8^+^ T cell responses in IFNAR^−/−^ recipients, viral control was severely impaired; LCMV titers in IFNAR^−/−^ recipients were at least 100,000-fold more compared to +/+ mice at day 5 after challenge (**Figure 9E**). LCMV-challenged IFNAR^−/−^ continued to harbor high levels of virus at least until day 8 after challenge. We were unable to assess viral clearance thereafter because P14 cell-transferred LCMV-challenged IFNAR^−/−^ mice succumbed to immunopathology within day 11 after challenge. Thus, Type I IFNs are essential for LCMV control during a secondary LCMV infection.

### Secondary response against vaccinia virus in PKR^−/−^ and IFNAR^−/−^ mice

**Figure 10:**
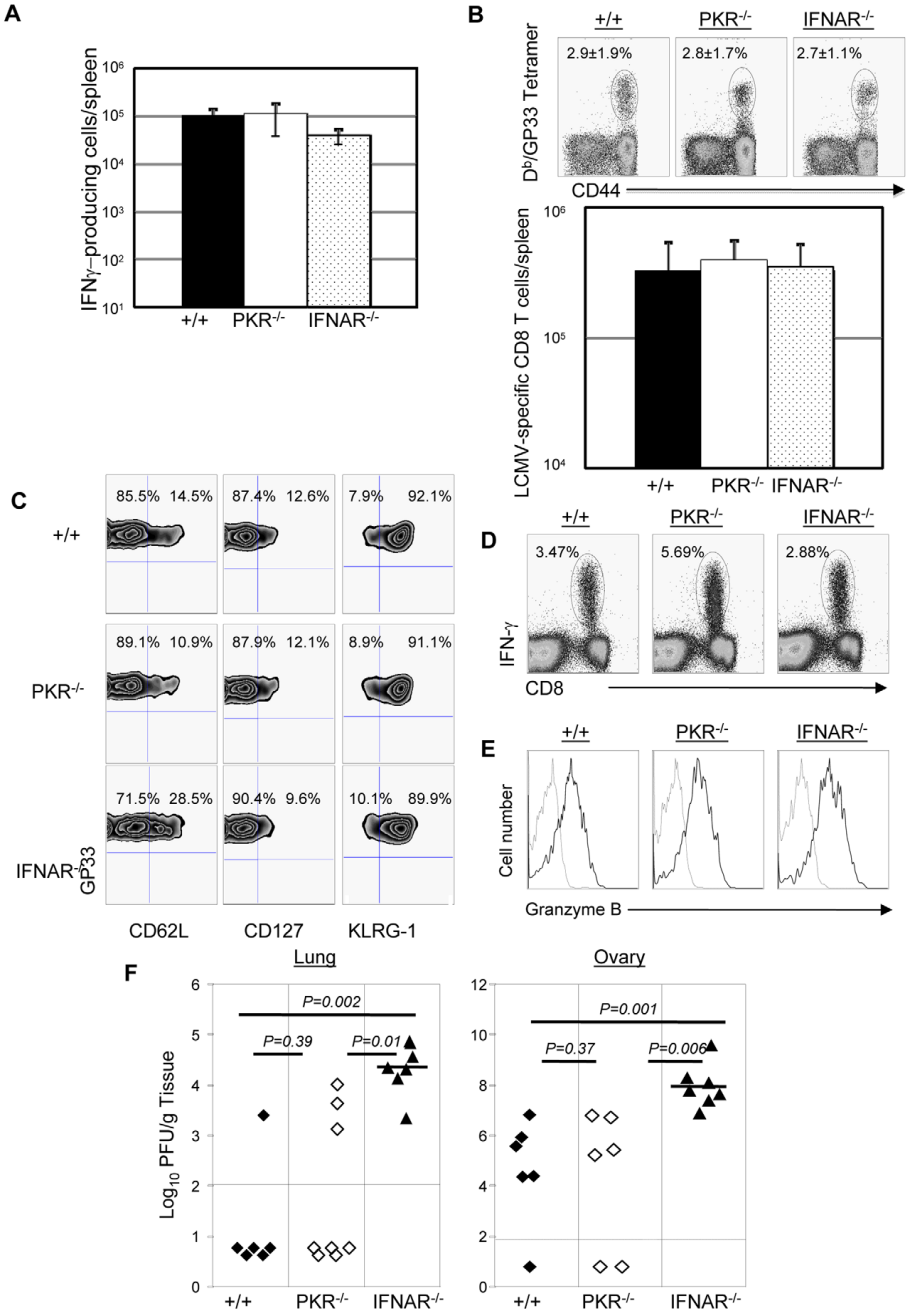
Secondary expansion of GP33-specific CD8^+^ T cells and protection against vaccinia virus in +/+, PKR^−/−^, and IFNAR^−/−^ mice. +/+, PKR^−/−^, and IFNAR^−/−^ mice were immunized with rLM-GP33. **A**. GP33-specific memory CD8^+^ T cells in LM-GP33-immune +/+, PKR^−/−^ and IFNAR^−/−^ mice. At day 90 PI, the number of GP33-specific memory CD8 T cells in spleen was quantitated by intracellular cytokine staining. **B**. Secondary expansion of GP33-specific CD8^+^ T cells in spleen of +/+, PKR^−/−^, and IFNRA^−/−^ mice. At 90 days after immunization with LM-GP33, +/+, PKR^−/−^ and IFNAR^−/−^ mice were challenged with recombinant vaccinia virus that expresses the glycoprotein of LCMV (VV-GP). Secondary expansion of GP33-specific CD8^+^ T cells was assessed in spleen five days after VV-GP challenge. Splenocytes were stained with anti-CD8, anti-CD44, and D^b^/GP33 MHC I tetramers. Dot plots in **B** are gated on total CD8^+^ T cells, and the numbers are the percentages of tetramer positive cells among total CD8^+^ T cells. **C**. Splenocytes from +/+, PKR^−/−^ or IFNAR^−/−^ recipients were stained with anti-CD8, anti-CD62L, anti-CD127, anti-KLRG-1, and D^b^/GP33 tetramers. Dot plots are gated on tetramer-binding CD8^+^ T cells, and the numbers are the percentages of cells in respective quadrants. **D**. Antigen-triggered IFN-γ production by CD8^+^ T cells from +/+, PKR^−/−^ or IFNAR^−/−^ mice; dot plots are gated on total splenocytes, and the numbers are the percentage of IFN-γ producing cells amongst splenocytes. **E**. Granzyme B expression in GP33-specific CD8^+^ T cells from +/+, PKR^−/−^, or IFNAR^−/−^ mice were measured by intracellular staining for granzyme B; histograms are gated on D^b^/GP33- tetramer-binding CD8^+^ T cells. Dotted and solid lines show staining with isotype control and anti-granzyme B antibodies respectively. **F**. VV-GP titers in lung and ovary from +/+, PKR^−/−^ or IFNAR^−/−^ mice; each symbol represents data from individual mice. Data is representative of or derived from two independent experiments, 3 to 4 mice per group.

Results presented in **Figures 4**, **5**, **8** and **9** clearly demonstrated the importance of PKR and Type I IFNs in controlling LCMV replication during a secondary CD8^+^ T cell response. Primary control of vaccinia virus requires Type I IFNs, but it is not known whether secondary CD8^+^ T cell memory-dependent control of vaccinia virus requires PKR and/or Type I IFNs [37], [38]. Therefore, we immunized groups of +/+, PKR^−/−^, and IFNAR^−/−^ mice with rLM-GP33. At day 90 after rLM-GP33 immunization (**Figure 10A**), the number of GP33-specific memory CD8+ T cells in PKR^−/−^ and IFNRA^−/−^ mice was comparable to those in +/+ mice. Next, rLM-GP33-immune +/+, PKR^−/−^, and IFNRA^−/−^ mice were challenged with recombinant vaccinia virus that expresses the glycoprotein of LCMV (VV-GP). At 5 days after challenge, the expansion of GP33-specific CD8^+^ T cells was assessed in the spleen. **Figures 10B** show that the total number of GP33-specific CD8^+^ T cells in PKR^−/−^ and IFNAR^−/−^ mice was comparable to those in +/+ mice. Additionally, the effector phenotype of GP33-specific CD8^+^ T cell in PKR^−/−^ and IFNAR^−/−^ mice was similar to those in +/+ mice (CD62L^low^, CD127^low^, and KLRG-1^high^) (**Figure 10C**). Furthermore, GP33-specific CD8^+^ T cells in PKR^−/−^ and IFNAR^−/−^ mice were capable of cytokine production and expressed high levels of granzyme B (**Figure 10D and 10E**). PKR deficiency alone did not appear to affect VV-GP control in lung and ovary. By contrast, all IFNAR^−/−^ mice uniformly exhibited impaired VV-GP control in both lung and ovary (**Figure 10F**). These data suggested that secondary control of vaccinia virus requires Type I IFNs, and the dependency of viral control on PKR and/or Type I IFNs is virus dependent.

## Discussion

The immune response to infection is divided into 2 phases: the early innate immune response and the later adaptive immune response. The mechanisms of innate immunity provide incomplete protection, but keep the pathogen in check until the more definitive adaptive immunity develops. In recent years, there is increased realization that mechanisms of innate immunity play a key role in both the induction and effector phases of adaptive immunity. In viral infections, Type I IFNs play a non-redundant role in early innate immunity, and in induction of the anti-viral T cell response [3], [5], [6], [37]. The IFN-inducible protein kinase PKR is a unique, multifunctional molecule that is known to act as a PRR for dsRNA and to mediate several of the anti-viral effects of Type I IFNs [9], [39], [40]. Additionally, PKR has been reported to regulate cellular proliferation, differentiation, and apoptosis [21], [41], [42], [43]. The non-redundant in vivo role of PKR as an innate anti-viral defense mechanism or as a regulator of virus-specific T cell response has not been examined. In the present study, we have dissected the importance of PKR in Type I IFN-dependent T cell regulation and cellular anti-viral defense during primary and secondary immune responses. Our studies document that Type I IFNs might exert their anti-viral and T cell regulatory effects by stimulating cellular pathways, which are dependent and independent of PKR respectively. We also provide strong evidence that presence of CD8^+^ T cell memory and accelerated generation of secondary effectors is insufficient to provide effective protective immunity to re-infection without the aid of innate effectors PKR and Type I IFNs. These findings have implications in understanding virus-immune system interactions and immune correlates of anti-viral protective immunity.

Upon infection with LCMV, both wild type and PKR^−/−^ mice developed potent and functional CD8^+^ T cell response, but only wild type mice were able to control viral replication by day 8 PI. Indeed, the expansion of virus-specific CD8^+^ T cells in PKR^−/−^ mice was reproducibly ∼2-fold higher compared to PKR^+/+^ mice. The increased expansion of CD8^+^ T cells in PKR^−/−^ mice was not likely due to higher viral load because during a chronic LCMV infection with clone 13 strain, under conditions of similar viral load, the expansion of CD8^+^ T cell was higher in PKR^−/−^ mice than in +/+ mice (data not shown). Nevertheless, our data are consistent with a previous report that PKR downregulates CD8^+^ T cell response in vivo in a delayed hypersensitivity model [20]. Previous work has shown that direct effects of Type I IFNs are required for optimal clonal expansion, survival, and differentiation of effector CD8+ T cells during an acute LCMV infection [6]. Our studies show that dependency of CD8^+^ T cells on Type I IFNs for clonal expansion during an acute LCMV infection might not include the requirement for PKR.

Defective LCMV control in PKR^−/−^ mice cannot be attributed to functional defects in virus-specific CD8^+^ T cells because PKR deficiency did not affect either the MHC-restricted cell-mediated cytotoxicity or the ability of CD8^+^ T cells to produce effector cytokines like IFN-γ and TNF-α directly ex vivo. PKR has been reported to be required for apoptosis of immune cells like macrophages [19]. Therefore, it is possible that LCMV-infected cells in PKR^−/−^ mice are more resistant to lysis by effector CD8^+^ T cells. However, we find that defective LCMV clearance in PKR^−/−^ mice is not likely due to cellular resistance to lysis by CD8^+^ CTLs. Detailed analysis of the kinetics of LCMV replication indicated that infectious LCMV levels between days 3 and 5 PI was reduced by ∼10 fold in wild type mice but increased by ∼10 fold in PKR^−/−^ mice between days 3 and 5 PI. This resulted in a ∼100 fold higher viral titer in PKR^−/−^ mice compared to PKR^+/+^ mice at day 5 PI. Loss of viral control between days 3 and 5 PI in PKR^−/−^ mice was similar to those in RAG1^−/−^ mice, which lack both B and T cells. These data indicated that delayed development of CD8^+^ T cell responses in PKR^−/−^ mice might underlie enhanced viral titers between days 3 and 5 PI. However, CD8^+^ T cell responses to LCMV in PKR^−/−^ mice at day 5 PI were indeed greater than in +/+ mice. These findings showed that defective viral control in PKR^−/−^ mice couldn't be linked to delayed emergence of anti-viral CD8^+^ T cell response. Based on these results, we propose that CD8^+^ T cell-dependent viral clearance early in the infection requires PKR activity in infected cells. Further studies are warranted to identify the PKR-dependent anti-viral effector functions of CD8^+^ T cells.

As mentioned elsewhere, PKR functions as a PRR for viral dsRNA and ssRNA containing 5′- triphosphate [44]. Interaction with viral RNA leads to activation of PKR, which in turn regulates various signaling pathways including induction of cytokines like Type I IFNs. However, PKR deficiency did not affect production of IFN-α or the activation of NK cells. One of the critical consequences of PKR activation is the inhibition of translation, which is considered a key step in viral clearance. In our studies, higher viral titers were attained upon infection of PKR-deficient BMDCs with LCMV, as compared to wild type BMDCs. Therefore, we propose that PKR^−/−^ BMDCs have an intrinsic defect in controlling LCMV replication. Apart from increased virus production, PKR-deficient BMDCs showed less sustained Type I IFN-induced suppression of LCMV replication in vitro. Based on these findings, we propose that: 1) LCMV control by innate defense mechanisms is at least in part dependent upon PKR; 2) sustained suppression of LCMV replication by Type I IFNs might include induction of PKR-dependent antiviral effects. By contrast to the anti-viral effects, the effects of Type I IFNs on CD8^+^ T cells appear to be independent of PKR. Based on these findings, we propose that distinct signaling pathways might participate in mediating diverse functions of Type I IFNs.

It is well established that potent CD8^+^ T cell memory is necessary and sufficient to protect against an acute or persistent LCMV infection [28]. However, it is unknown whether innate immune mechanisms are important in viral control during the secondary CD8^+^ T cell responses. Immunocompetent mice immunized with recombinant *Listeria monocytogenes* that expresses the immunodominant LCMV CD8^+^ T cell epitope GP33 (rLM-GP33), is known to confer effective protective immunity against LCMV [45]. We show that PKR^−/−^ and IFNAR^−/−^ mice immunized with rLM-GP33 mounted potent secondary CD8^+^ T cell responses upon challenge with LCMV. Surprisingly, despite the normal induction of secondary CTL responses, both PKR^−/−^ and IFNAR^−/−^ mice are unable to effectively control LCMV. Impaired control of LCMV in rLM-GP33-immunized PKR^−/−^ mice is likely due to a loss of PKR-dependent antiviral mechanism in virus-infected cells because adoptively transferred wild type TCR tg LCMV-specific memory CD8^+^ T cells conferred protective immunity in wild type mice, but not in PKR^−/−^ or Type I IFN receptor deficient (IFNAR^−/−^) mice. Thus, PKR and IFNAR signaling are required in infected cells, rather than in CD8^+^ T cells for control of LCMV infection during secondary CD8^+^ response. The number of memory CD8^+^ T cells present at the time of re-infection or challenge might regulate the role of PKR or Type I IFNs in viral control during a secondary response. Nevertheless, in the physiological setting of immunization with a live LM vaccine, CD8^+^ T cell memory is unable to effectively control LCMV in the absence of PKR or Type I IFNs.

It is worth noting that LCMV titers in IFNAR^−/−^ mice were considerably greater than in PKR^−/−^ mice, which suggests the involvement of PKR-independent Type I IFN-triggered antiviral mechanisms in LCMV control. It would be interesting to examine protective immunity in LCMV in triple knockout mice lacking all the major ISGs of Type I IFNs, PKR, RNase L, and Mx-1 [46].

As discussed before, GP33-specific CD8^+^ memory T cells in LM/GP33-immune IFNRA^−/−^ mice showed normal expansion and differentiation into effector cells, upon challenge with LCMV. Interestingly, GP33-specific effector CD8^+^ T cells in wild type but not IFNRA^−/−^ mice downregulated cell surface expression of CD127, the IL-7 receptor. The inability of INFRA^−/−^ deficient CD8^+^ T cells to downregulate CD127 expression could not be linked to lack of direct effects of Type I IFNs because, adoptively transferred donor IFNRA-sufficient GP33-specific memory P14 CD8^+^ T cells also failed to downregulate CD127 expression in LCMV-infected IFNRA^−/−^ mice. These findings suggest that Type I IFNs regulate CD127 expression on effector CD8^+^ T cells by indirect effects. It has been reported that cytokines like IL-6 and IL-15 downregulate CD127 expression on CD8^+^ T cells [47], and importantly these cytokines are induced by Type I IFNs [48], [49], [50], [51], [52], [53]. Therefore, we speculate that defects in induction of cytokines like IL-15 and/or IL-6 might impede downregulation of CD127 expression on effector CD8^+^ T cells in IFNRA^−/−^ mice. Notably, unlike challenge with LCMV, loss of IFNRA signaling was not required for CD127 downregulation on effector CD8^+^ T cells upon challenge with vaccinia virus. These findings suggest that infection with vaccinia virus but not LCMV might induce factor(s) that downregulates CD127 expression in the apparent absence of Type I IFNs, which in turn could be linked to differences in pathogenesis including cell tropism of the virus and the assortment of host responses elicited during infection.

Similar to LCMV infection, Type I IFNs play a critical role in control of vaccinia virus infection; IFNAR^−/−^ mice are highly susceptible to vaccinia virus infection [25], [26], [54]. Interestingly, however, unlike in an LCMV infection, Type I IFN signaling but not PKR is required for control of vaccinia virus during secondary CTL responses. These findings suggested that PKR-independent mechanisms are important in vaccinia control by Type I IFNs. The differential requirement for PKR in control of LCMV and vaccinia virus is unknown. Only vaccinia virus and not LCMV is known to encode proteins that antagonize the activation and/or cellular function of PKR [14], [55]. Therefore, the antiviral actions of PKR are likely to be diminished in vaccinia-infected cells but preserved in LCMV-infected cells. As a result, PKR deficiency would be expected to have minimal effects on vaccinia virus replication. It has to be noted that vaccinia virus also encodes proteins to neutralize PKR-independent IFN actions [14], [55], which are obviously less effective in fully circumventing the anti-viral effects of Type I IFNs, because deficiency of Type I IFNs leads to impaired viral control [25], [26], [54].

In summary, we present new findings in this manuscript that further our understanding of the cooperative interactions between mechanisms of innate and adaptive immunity from the standpoint of viral control and elicitation of virus-specific CD8^+^ T cell responses. First, we document that requirement for cellular PKR activity could be a distinguishing feature between Type I IFN actions in mediating viral control versus CD8^+^ T cell activation during an acute LCMV infection. Based on our studies we propose that PKR controls LCMV replication by at least three mechanisms: 1) augmenting innate antiviral mechanisms in infected cells by promoting the expression of ISGs; 2) sustain Type I IFN-induced antiviral actions in infected cells; 3) mediate a mechanism of CD8^+^ T cell-dependent viral control. Finally, we provide strong evidence that vaccine-induced immunological memory is necessary but not sufficient to provide protective immunity against viral infection in the absence of PKR or Type I IFNs. These findings have significant implications in understanding viral pathogenesis and immune correlates of protection against viruses.

## Materials and Methods

### Mice

C57BL/6 mice were purchased from the National Cancer Institute (Frederick, MD). RAG1^−/−^ mice were purchased from the Jackson Laboratory (Bar Harbor, ME). Generation of PKR deficient mice (PKR^−/−^) on the C57BL/6 background were described previously [56]. The IFNRA^−/−^ mice on the C57BL/6 background [6] were provided by Dr. Kaja Murali-Krishna (University of Washington, Seattle). All mice were used at 6-8 weeks of age according to the protocol V847 approved by the University of Wisconsin School of Veterinary Medicine Institutional Animal Care and Use Committee (IACUC). The animal committee mandates that institutions and individuals using animals for research, teaching, and/or testing must acknowledge and accept both legal and ethical responsibility for the animals under their care, as specified in the Animal Welfare Act (AWA) and associated Animal Welfare Regulations (AWRs) and Public Health Service (PHS) Policy.

### Virus

Mice were infected intraperitoneally with 2×10^5^ PFU of the Armstrong stain of LCMV [57]. Infectious LCMV in the tissues was quantitated by a plaque assay using Vero cells [57]. The recombinant vaccinia virus VV-GP that expresses the glycoprotein of LCMV was provided by Lindsay Whitton (Scripps Research Institute, La Jolla, CA) [58]. Mice were challenged with 2×10^6^ PFU of VV-GP by i.p. injection. VV-GP was quantitated by plaque assay using CV-1 cells [58].

### *Listeria monocytogenes*

Mice were infected with 5×10^4^ cfu of recombinant *Listeria monocytogenes* that expresses the glycoprotein 33–41 epitope of LCMV (rLM-GP33) by intravenous injection [45]. Bacterial load in tissues was quantitated by plating tissue homogenates on brain-heart infusion agar plates [45].

### Cell surface staining and flow cytometry

The preparation and use of MHC I tetramers specific to the LCMV epitopes nucleoprotein 396–404 (NP396) and glycoprotein 33–41 (GP33) have been described previously [59]. Splenocytes were stained with fluorescent conjugated anti-CD8 (Clone 53–6.7), anti-CD44 (Clone IM-7), and MHC I tetramers at 4 C for 1 h. Cells were also stained with antibodies against CD43 (Clone 1B11), CD62L (Clone MEL-14), CD127 (Clone A7R34), and CD69 (Clone H1.2F3) in conjunction with MHC I tetramers. In some experiments splenocytes were stained with anti-Ly5.1 (Clone A20) and anti- KLRG-1 (Clone 2F1) antibodies. To analyze expression of Type I IFN receptors, peripheral blood mononuclear cells were stained with anti-IFNAR antibodies (Clone MAR1-5A3; Leinco Technologies, MO). Following staining, cells were fixed in 2% paraformaldehyde and samples were acquired with a FACSCalibur flow cytometer (BD Biosciences, La Jolla, CA).

### Intracellular staining for cytokines and granzyme B

Intracellular staining for IFN-γ and TNF-α was performed as described previously [59]. Briefly, freshly explanted splenocytes (10^6^ cells/well) were cultured with or without the LCMV epitope peptides (0.1 ug/ml) in the presence of brefeldin A (Golgistop; Pharmingen) and human recombinant interleukin-2 (10 U/well) in 96-well flat-bottom plates. After 6 hours of in vitro stimulation, cells were stained for surface CD8 and intracellular IFN-γ (Clone XMG1.2) and TNF-α (Clone MP6-XT22) using the cytofix/cytoperm intracellular staining kit (BD Pharmingen). To stain for granzyme B, splenocytes were first incubated with MHC I tetramers, followed by permeabilization and staining for intracellular granzyme B (Clone GB12; Caltag).

### ELISA

IFN-α level in the mouse serum was measured with ELISA kit (R & D Systems, Minneapolis, MN) as per the recommendations of the manufacturer.

### LCMV growth curve in BMDCs with or without treatment with IFN-β

BMDCs were generated as described before [60]. Briefly, bone marrow cells were collected from the tibias and femurs of +/+ or PKR^−/−^ mice. After RBC lysis, cells were resuspended at a concentration of 10^6^/ml and plated in 6-well plates in RPMI 1640 media containing 10% FBS and 20 ng/ml of murine GM-CSF (PEPROTECH Inc, NJ). DCs were cultured for 6 days at 37C in 5% CO_2_ and half of the media containing GM-CSF was replaced on days 2 and 4. On day 6, DCs were harvested for treatment with IFN-β and infection with LCMV as follows. BMDCs (10^5^/ml) were plated onto 48-well plates and left untreated or treated with IFN-β (R & D Systems, Minneapolis, MN) for 20 to 24 hours before LCMV infection (MOI = 0.01). The supernatant from each well were collected for plaque assay.

### RT-PCR

BMDCs described above were left untreated or treated with 1000 U/ml of IFN-β for 6 hours. Total RNA was extracted from the BMDCs by using an RNA extraction kit (RNAqueous; Ambion, Austin, TX). RNA was reverse transcribed to cDNA by using Moloney murine leukemia virus reverse transcriptase from Invitrogen (Carlsbad, CA). Equivalent amounts of cDNA (as determined by 18S rRNA measurements by quantitative PCR) were amplified in 35 cycles of PCR with SYBR green (Applied Biosystems, Foster City, CA) by using primers designed for IRF-1, IRF-3, IRF-5, and IRF-7, and analyzed by Applied Biosystems 7300 Real Time PCR System (Applied Biosystems, Foster City, CA). Primer sets for IRF-1 were CAGCACTGTCACCGTGTGTCGT (forward) and GCGGCTTCGGAGGTGGAA (reverse), IRF-3 were TGGGCAGCACAGCTGACATGA (forward) and GCCCATTGCCCAGCCCTT (reverse), IRF-5 were CCTGCGCTGTGCCCTTAACA (forward) and TGTGGGAGCAGGGCCGTT (reverse) and IRF-7 were GCCTTGGGTTCCTGGATGTGA (forward) and TGGGGCCATGGGGCTGTA (reverse). Relative expression ratio of the target gene was determined based on 18S rRNA expression by using the following primer sets: CGCCGCTAGAGGTGAAATTCT (forward) and CGAACCTCCGACTTTCGTTCT (reverse).

### NK cell assay

YAC-1 target cells were obtained from a three day culture and labeled with ^51^Cr. Splenocytes obtained from mice, infected with LCMV three days before, were used as source of effector cells. Effector and target cells were mixed at different ratios and the supernatants were harvested after 5 hours. ^51^Cr release was counted by a gamma counter and the percent lysis of target cells by effector cells was calculated (Packard Instrument Company, Meriden, CT).

### Cytotoxicity assay

MHC class I restricted LCMV-specific cytotoxic activity in the spleens was measured ex vivo by a standard ^51^Cr-release assay using syngeneic LCMV peptide-pulsed MC57 cells as target cells [57]. ^51^Cr-labeled MC57 target cells were pulsed with LCMV epitope peptides and mixed with effector (splenocytes) cells from +/+ or PKR^−/−^ mice at different indicated ratios. In some experiments, ^51^Cr-labeled LCMV-infected primary BMDCs derived from +/+ or PKR^−/−^ mice were mixed with splenocytes from B6 mice infected with LCMV. After 5 hours, ^51^Cr release was quantified and the percent killing was calculated.

### In vivo CTL assay

The MHC I restricted cytotoxic activity was assessed in vivo as described previously [61]. Briefly, splenocytes from naïve +/+ or PKR^−/−^ mice were labeled with either 0.1 µM or 2 µM of CFSE (Molecular Probes, Eugene, OR). Splenocytes labeled with 2 µM CFSE (CFSE^high^) were pulsed with GP33 peptide (1 µg/ml) for 1 hour at 37C, whereas splenocytes labeled with 0.1 µM CFSE (CFSE^low^) were not pulsed with peptide. Equal proportions of peptide-pulsed and unpulsed CFSE-labeled splenocytes were mixed together and transferred to naïve or LCMV Arm-infected mice by intravenous injection. Five hours later, the recipient mice were euthanized and recovery of peptide-pulsed and unpulsed target cells in spleens was quantified by flow cytometry. The percent killing was calculated as follows: 100- {[(% peptide pulsed in infected/% unpulsed in infected)/(% peptide pulsed in uninfected/% unpulsed in uninfected)] X100}.

### Generation and adoptive transfer of P14 memory CD8^+^ T cells

2×10^5^ naïve Ly5.1/P14 CD8^+^ T cells were adoptively transferred into congenic C57BL/6/Ly5.2 mice. Twenty-four hours after cell transfer, mice were infected with LCMV as above. At least >100 days after infection, T cells were purified from the spleens using T cell enrichment columns (R&D systems, Minneapolis, MN) for adoptive transfer. Purified T cells containing 2×10^5^ memory P14 TCR tg CD8^+^ T cells were adoptively transferred into +/+, PKR^−/−^, or IFNRA^−/−^ mice, which were subsequently challenged with LCMV.

### Immunofluorescent staining of tissues

Tissues were embedded in OCT compound and snap frozen with liquid nitrogen. Frozen sections were cut with cryostat and fixed with acetone at 4C, dried and stained with antibody conjugated with fluorescent dye Alexa 568.

### Statistical analysis

The commercially available software (SYSTAT [Chicago, IL], version 10.2) was used to analyze data. To analyze for differences between groups, unpaired t test was performed at 95% confidence interval. In some analyses, differences between groups were compared by testing for the equality of the proportions of detectable viral titers. Some statistical analyses were done using the Prizm software also.

## Supporting Information

Figure S1 LCMV clearance in RAG1-deficient mice. Groups of wild type +/+ and RAG1-deficient (RAG1^−/−^) mice were infected with LCMV, and viral titers were quantitated by plaque assay; each symbol represents data from an individual mouse. Data is from one of two independent experiments.
(0.05 MB TIF)

Figure S2 CD8^+^ T cell responses to LCMV in PKR-deficient mice (day 5 PI). Groups of wild +/+ and PKR^−/−^ mice were infected with LCMV, and CD8^+^ T cells that are specific to the two immunodominant epitopes were quantitated in the spleen by using MHC I tetramers. Data are the mean of 3–4 mice/group and representative of two independent experiments.
(0.03 MB TIF)

Figure S3 IFN-β-induced suppression of LCMV replication in +/+ and PKR^−/−^ BMDCs. As described for Figure 3, BMDCs from +/+ and PKR^−/−^ mice were left untreated or pretreated with the indicated levels of IFN-β for 20 to 24 hours, then infected with LCMV at 0.01 MOI. The supernatants of triplicate cultures were collected at the indicated time points and viral titers were determined by plaque assay. The percent reduction of viral titer following treatment with IFN-β, as compared to no treatment was calculated for each dose at all time points.
(3.73 MB TIF)

Figure S4 Cell surface expression of Type I IFN receptors on PKR^−/−^ cells. Peripheral blood mononuclear cells from +/+, IFNRA^−/−^, and PKR^−/−^ mice were stained with anti-CD8, anti-CD4, anti-B220, and anti-Type I IFN receptor antibodies; cells from IFNRA^−/−^ are used as negative controls. Following staining, the cell surface expression of Type I IFN receptor on CD8^+^, CD4^+^, and B220^+^ cells was assessed by flow cytometry. The FACS histograms showing staining for the Type I IFN receptor, are gated on the indicated cell population. Data shown are from one of two independent experiments.
(0.05 MB TIF)

Figure S5 Effector and central memory CD8^+^ T cells in LCMV-immune PKR^−/−^ mice. Groups of +/+ and PKR^−/−^ mice were infected with LCMV. At 90 days after infection, splenocytes were stained with anti-CD8, D^b^/MHC I tetramers, and anti-CD62L. The cell surface expression of CD62L on tetramer-binding CD8^+^ T cells was assessed by flow cytometry. The data shows the percentages of CD62L^lo^ (effector memory subset) cells amongst epitope-specific CD8^+^ T cells. Data are from one of two independent analyses of LCMV-specific memory CD8^+^ T cells in PKR^−/−^ mice.
(0.03 MB TIF)
