# Supplementary figures and images for: Role of PKR and Type I IFNs in Viral Control during Primary and Secondary Infection
Source: PLoS Pathog. 2010 Jun 24;6(6):e1000966. doi: 10.1371/journal.ppat.1000966 (PMC2891951; doi:10.1371/journal.ppat.1000966)

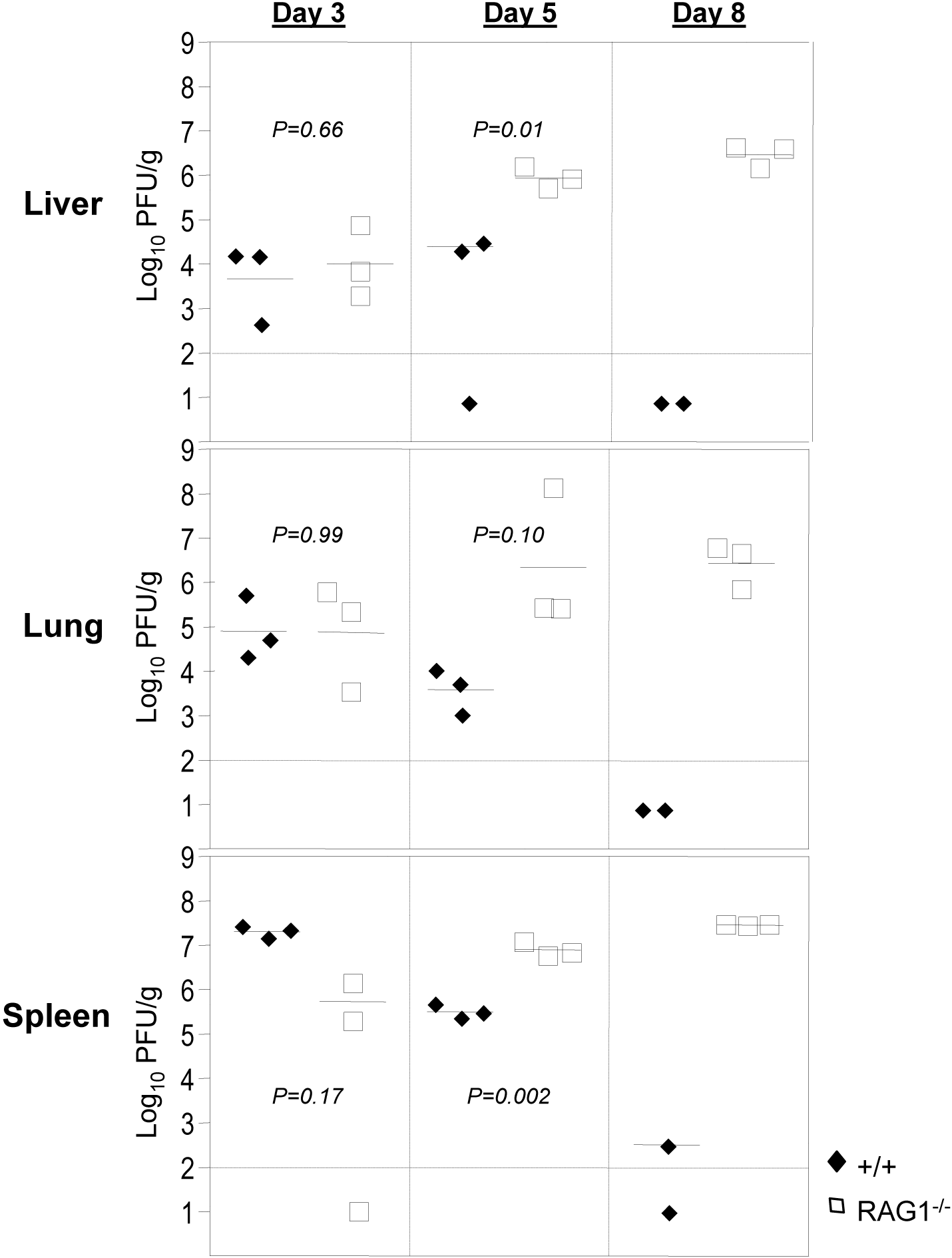

Supplement: Figure S1 — LCMV clearance in RAG1-deficient mice. Groups of wild type +/+ and RAG1-deficient (RAG1−/−) mice were infected with LCMV, and viral titers were quantitated by plaque assay; each symbol represents data from an individual mouse. Data is from one of two independent experiments. (0.05 MB TIF) [file ppat.1000966.s001.tif]

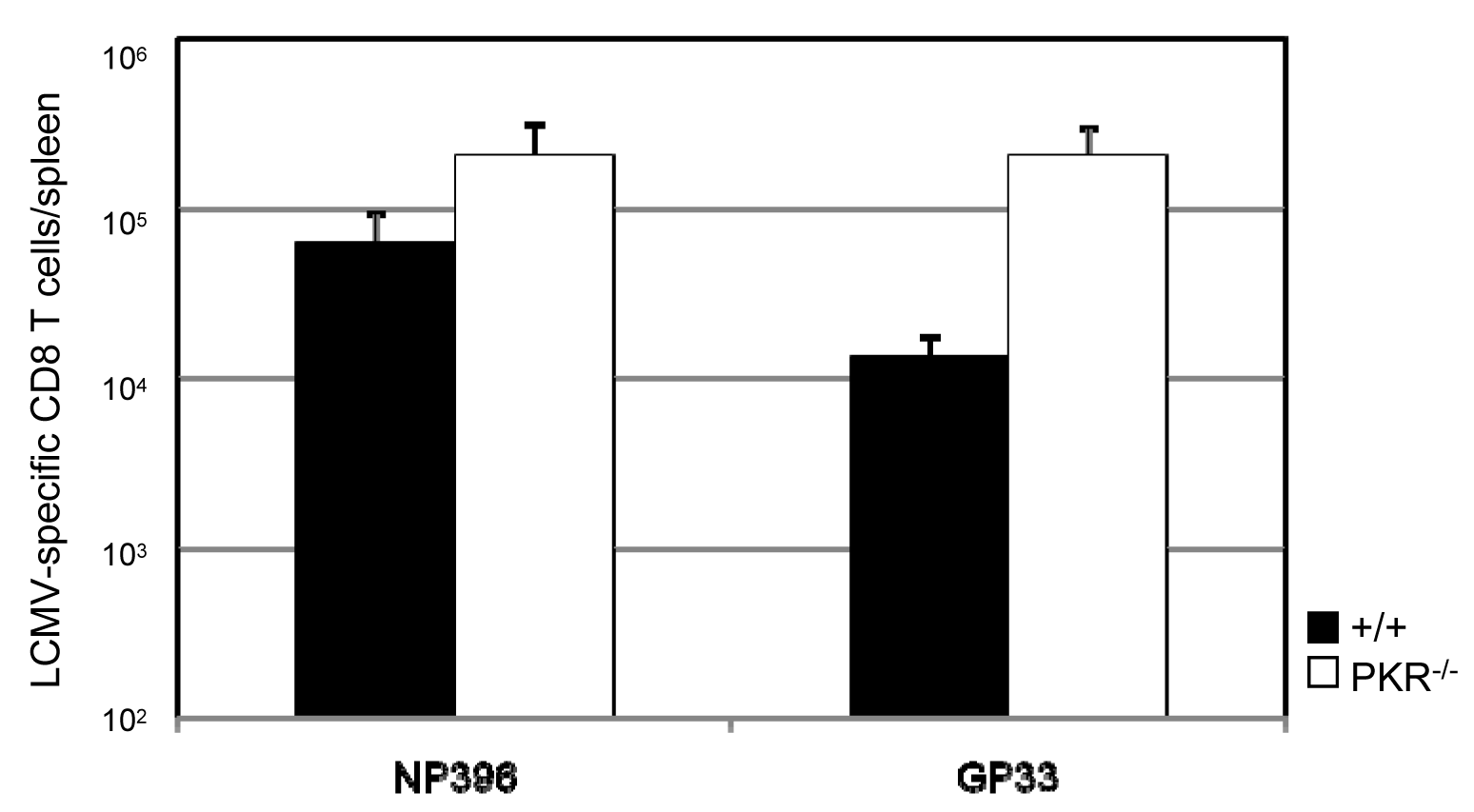

Supplement: Figure S2 — CD8+ T cell responses to LCMV in PKR-deficient mice (day 5 PI). Groups of wild +/+ and PKR−/− mice were infected with LCMV, and CD8+ T cells that are specific to the two immunodominant epitopes were quantitated in the spleen by using MHC I tetramers. Data are the mean of 3–4 mice/group and representative of two independent experiments. (0.03 MB TIF) [file ppat.1000966.s002.tif]

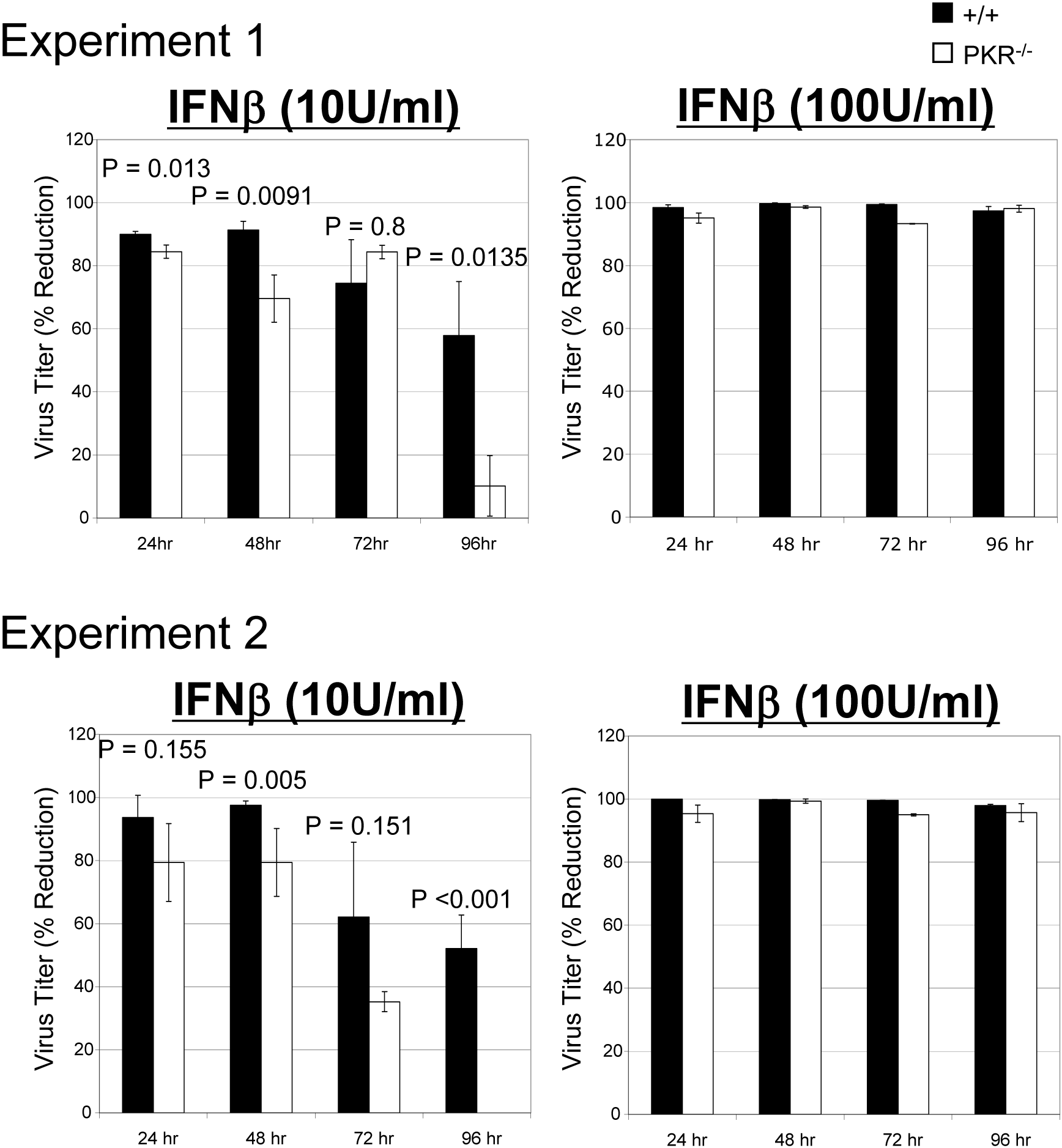

Supplement: Figure S3 — IFN-β-induced suppression of LCMV replication in +/+ and PKR−/− BMDCs. As described for Figure 3, BMDCs from +/+ and PKR−/− mice were left untreated or pretreated with the indicated levels of IFN-β for 20 to 24 hours, then infected with LCMV at 0.01 MOI. The supernatants of triplicate cultures were collected at the indicated time points and viral titers were determined by plaque assay. The percent reduction of viral titer following treatment with IFN-β, as compared to no treatment was calculated for each dose at all time points. (3.73 MB TIF) [file ppat.1000966.s003.tif]

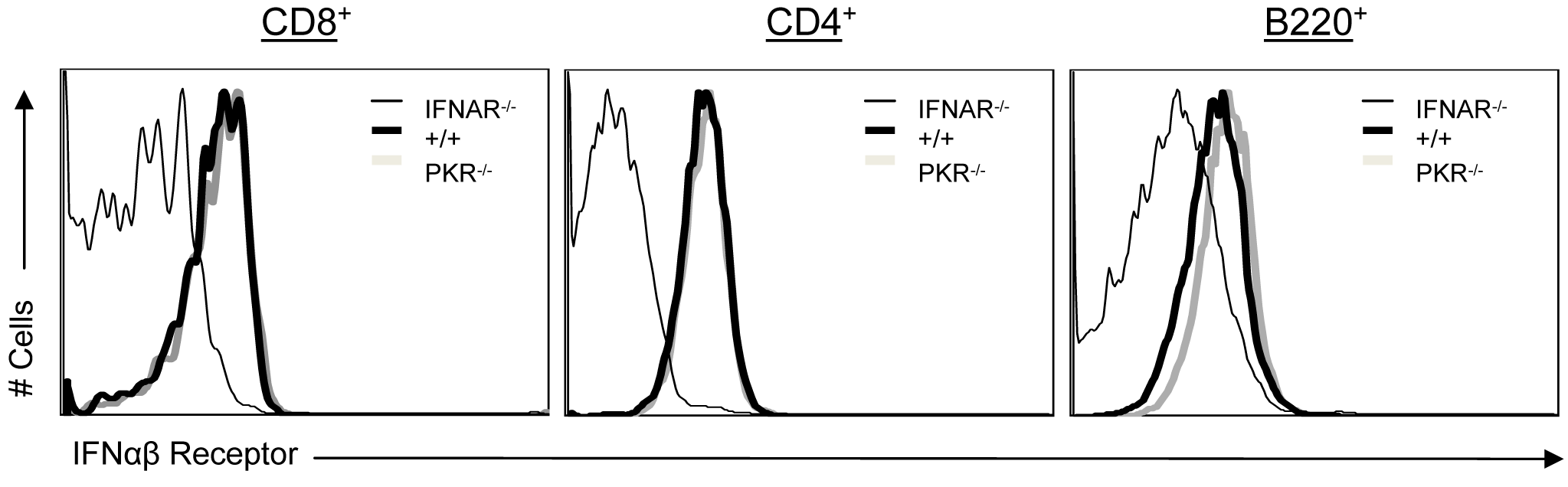

Supplement: Figure S4 — Cell surface expression of Type I IFN receptors on PKR−/− cells. Peripheral blood mononuclear cells from +/+, IFNRA−/−, and PKR−/− mice were stained with anti-CD8, anti-CD4, anti-B220, and anti-Type I IFN receptor antibodies; cells from IFNRA−/− are used as negative controls. Following staining, the cell surface expression of Type I IFN receptor on CD8+, CD4+, and B220+ cells was assessed by flow cytometry. The FACS histograms showing staining for the Type I IFN receptor, are gated on the indicated cell population. Data shown are from one of two independent experiments. (0.05 MB TIF) [file ppat.1000966.s004.tif]

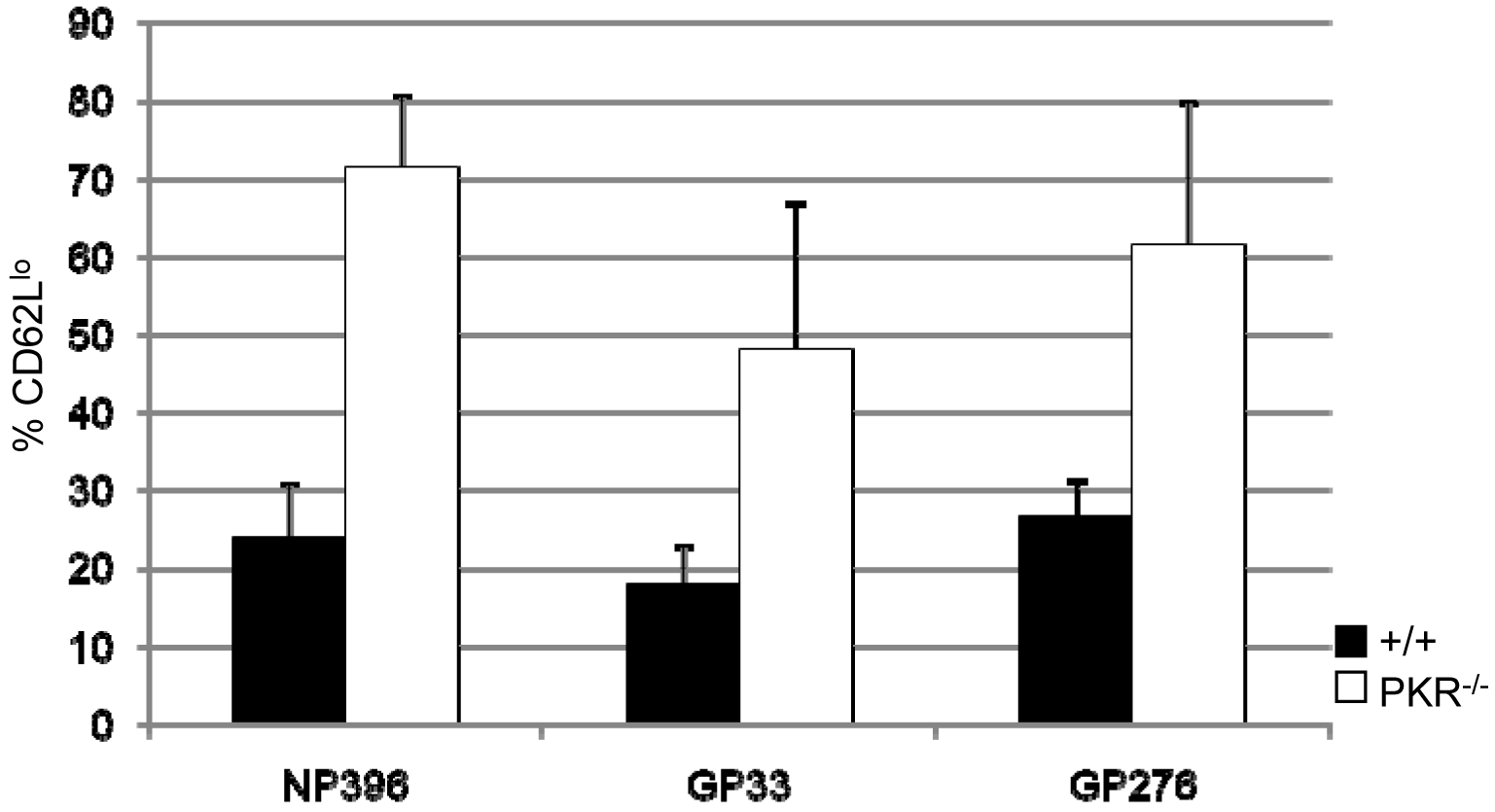

Supplement: Figure S5 — Effector and central memory CD8+ T cells in LCMV-immune PKR−/− mice. Groups of +/+ and PKR−/− mice were infected with LCMV. At 90 days after infection, splenocytes were stained with anti-CD8, Db/MHC I tetramers, and anti-CD62L. The cell surface expression of CD62L on tetramer-binding CD8+ T cells was assessed by flow cytometry. The data shows the percentages of CD62Llo (effector memory subset) cells amongst epitope-specific CD8+ T cells. Data are from one of two independent analyses of LCMV-specific memory CD8+ T cells in PKR−/− mice. (0.03 MB TIF) [file ppat.1000966.s005.tif]
